# Do Double-Hybrid Functionals Benefit from Regularization in the PT2 Term? Observations from an Extensive Benchmark

**DOI:** 10.1021/acs.jpclett.2c00718

**Published:** 2022-04-13

**Authors:** Golokesh Santra, Jan M. L. Martin

**Affiliations:** Department of Molecular Chemistry and Materials Science, Weizmann Institute of Science, 7610001 Reḥovot, Israel

## Abstract

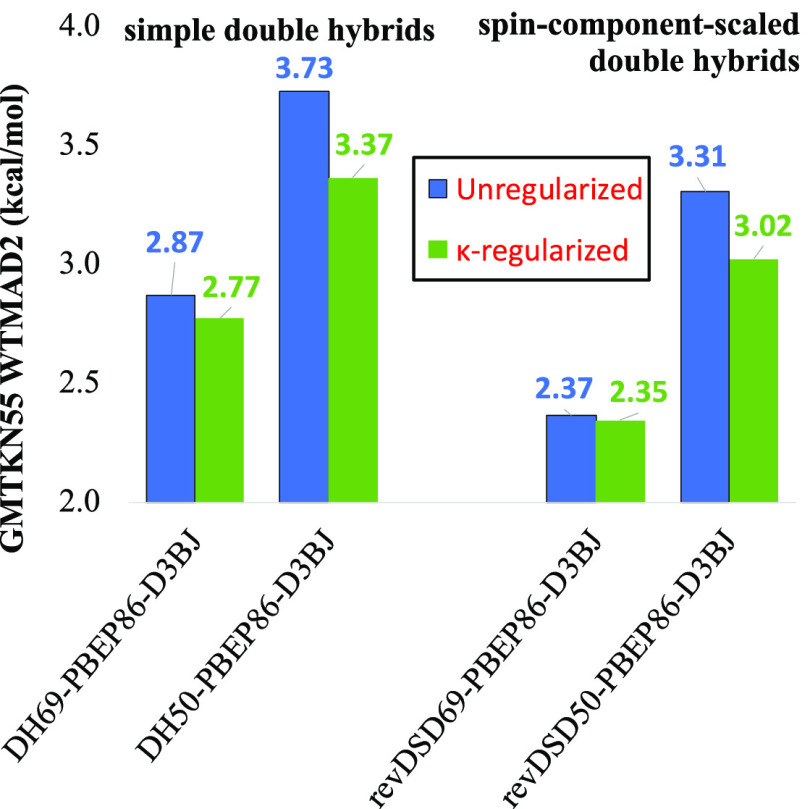

We put to the test a recent suggestion [Shee, J., et al. *J. Phys. Chem. Lett.***2021**, *12* (50), 12084–12097] that MP2 regularization might improve the performance of double-hybrid density functionals. Using the very large and chemically diverse GMTKN55 benchmark, we find that κ-regularization is indeed beneficial at lower percentages of Hartree–Fock exchange, especially if spin-component scaling is not applied [such as in B2GP-PLYP or ωB97M(2)]. This benefit dwindles for DSD and DOD functionals and vanishes entirely in the ∼70% HF exchange region optimal for them.

Double-hybrid density functional (DHDF) theory (for reviews, see refs (1−4)) represents a special case of fifth-rung functionals on “Jacob’s Ladder”^5^ (the fifth rung is where dependence on unoccupied orbitals enters). As such, DHDF theory resides on the seamline between density functional theory (DFT) and wave function approaches. DHDFs are the most accurate DFT methods available to date for main group energetics,^6−8^ transition metal catalysis,^9−13^ electronic excitation spectroscopy,^14−22^ external magnetic^23−26^ and electric field^27,28^ induced properties, and vibrational frequencies.^29^ Its reliance on a second-order perturbation theory term for part of the correlation energy creates an Achilles’ heel for molecules with small band gaps, due to the presence of orbital energy differences in the denominator. Using the spin–orbital notation, the MP2-like Görling–Levy^30^ nonlocal correlation term (GLPT2) has the following form:
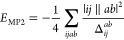
where the indices *i* and *j* refer to occupied orbitals and *a* and *b* to virtual orbitals, while the energy denominator Δ*_ij_^ab^* = ε_*a*_ + ε_*b*_ – ε*_i_* – ε_*j*_.

One remedy that has been proposed in the past, in the context of single-reference or multireference MP2, has been DCPT (degeneracy-corrected perturbation theory^31^). Another is regularization of the expression presented above to remove the singularity for Δ*_ij_^ab^* → 0. Stück and Head-Gordon^32^ proposed a simple level-shift regularizer (δ) in the context of ROOMP2^33−35^ (restricted orbital-optimized second-order perturbation theory). They found that this works well for single-bond breaking, but the required level shifts for multiple bonds were so large that they disrupted thermochemistry results.^36^ Lee and Head-Gordon^37^ have recently proposed two energy gap-dependent regularizers: σ and κ. Although these forms were developed initially with OOMP2 in mind, Head-Gordon and co-workers^38,39^ have shown that even without orbital optimization, σ- and κ-MP2 can achieve better accuracy than ordinary (unregularized) MP2 for both main group and transition metal thermochemistry, barrier heights, and noncovalent interactions. They also found that both sets of regularizers performed comparably; hence, we will focus here on only κ-regularized MP2 correlation, which has the following expression:
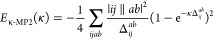
where κ is a fixed regularization parameter. In the large-κ or large-Δ*_ij_^ab^* limits, the regularization factor approaches unity, while in the small-κ or small-Δ*_ij_^ab^* limits, the corresponding term in the energy summation approaches zero, i.e., the Hartree–Fock energy is recovered.

Reference (37) states that “We are optimistic that this study could pave the way for future development of double-hybrid density functionals based on nonlocal correlation expressions that are more appropriate than conventional MP2 for large dispersion-bound systems and organometallic bonding, yet still free of self-correlation errors. κMP2, σ^2^MP2, and σMP2 are promising candidates in this regard.”

Such DHDFs as our own minimally empirical dispersion-corrected spin-component scaled families, e.g., revDSD,^37^ revDOD,^37^ and xDSD,^40^ Grimme’s PWPB95,^41^ or the more heavily parametrized ωB97M(2)^42^ range-separated DHDF reach accuracies approaching those of composite wave function approaches (see ref (43) for a head-to-head comparison). Yet there might still be room for further improvement, particularly in terms of resilience for systems with small band gaps, and hence significant type A static correlation (also known as absolute near-degeneracy correlation^44^).

In this letter, we will attempt to confirm or refute the conjecture presented above from ref (37), that is, to determine whether using spin-component-scaled κ-GLPT2 instead of unregularized (i.e., conventional) PT2 correlation can further improve the performance of DSD functionals. For the κ-regularized DSD functionals, the final energy has the following expression:
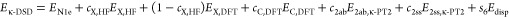
*E*_N1e_ stands for the sum of nuclear repulsion and one-electron energy terms. *E*_X,HF_ represents the exact exchange, and *c*_X,HF_ the corresponding coefficient. *E*_X,DFT_ is the exact exchange energy component from the semilocal generalized gradient approximation (GGA), and *c*_X,DFT_ the corresponding parameter. *E*_C,DFT_ represents the semilocal GGA correlation component, and *c*_C,DFT_ the coefficient for that energy part. *E*_2ab,κ-PT2_ and *E*_2ss,κ-PT2_ are the opposite-spin and same-spin κ-GLPT2 correlation energies, respectively, and their respective linear coefficients are *c*_2ab_ and *c*_2ss_. Finally, *E*_disp_ is a dispersion correction such as D3(BJ)^45−47^ with any associated adjustable parameters: in the work presented here (as in ref (48)), the nonlinear damping-function shape parameters are fixed at *a*_1_ = 0 and *a*_2_ = 5.5, and the higher-order coefficient is fixed at *s*_8_ = 0 (as we have found^48^ to be appropriate for double hybrids).

We have used the GMTKN55 benchmark suite^7^ (general main group thermochemistry, kinetics, and noncovalent interactions) throughout. It comprises 55 types of chemical problems, which can be further divided into five major subcategories: thermochemistry of small- and medium-sized molecules, barrier heights, large-molecule reactions, intermolecular interactions, and intramolecular interactions (or conformer energies). WTMAD2 (weighted mean absolute deviation) as defined in eq 2 of ref (7) has been used as our primary metric:
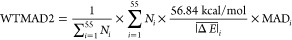
where  is the mean absolute value of all of the reference energies from *i* = 1 to 55, *N*_*i*_ is the number of systems in each subset, and MAD_*i*_ is the mean absolute difference between calculated and reference energies for each of the 55 subsets. For the details of all 55 subsets with proper references, see Table 1 of ref (7).

All electronic structure calculations were performed using the QCHEM 5.4^49^ package on the ChemFarm HPC cluster in the Faculty of Chemistry at the Weizmann Institute of Science. The Weigend–Ahlrichs def2-QZVPP^50^ basis set was used for all subsets except seven, for which we used the diffuse-function augmented variant def2-QZVPPD^51^ instead: the rare gas clusters RG18 and the six anion-containing subsets WATER27, IL16, G21EA, BH76, BH76RC, and AHB21. For the C60ISO and UPU23 subsets (which have comparatively small weights in WTMAD2), we settled for the def2-TZVPP basis set to reduce the computational cost. For the remaining 46 subsets, the def2-QZVPP^50^ basis set was used. The corresponding standard RI-MP2^52^ and Coulomb exchange fitting (RI-JK)^53^ basis sets were employed throughout to reduce the computational cost further. The large pruned integration grid SG-3 was used across the board.^54^ Generally, inner-shell orbitals were frozen, but in subsets where such orbitals come close enough to the valence shell to qualify as “honorary valence orbitals”,^55^ the same frozen-core settings were used as in refs (48), (56), and (57).

Similar to the unregularized (*x*)DSD functionals, one fully optimized κ-DSD also has six adjustable linear parameters: *c*_X,HF_, *c*_C,DFT_, *c*_2ab_, *c*_2ss_ (=*c*_2aa_ + *c*_2bb_), and for the D3(BJ) dispersion correction one prefactor *s*_6_ and one parameter *a*_2_ for the damping function (like in refs (58) and (59), we constrain *a*_1_ and *s*_8_ to zero). In addition, our new κ(*x*)DSD functionals also contain the PT2 regularization parameter κ.

Powell’s BOBYQA^60^ (bound optimization by quadratic approximation) derivative-free constrained optimizer together with in-house written scripts and Fortran programs were used to optimize all parameters.

To find our bearings, we first explore the effect of using κ-GLPT2 energies in the revDSD-PBEP86-D3BJ and xDSD_75_-PBEP86-D3BJ double hybrids using the “Diet-100” statistical reduction^61^ version of GMTKN55. The unregularized same-spin and opposite-spin PT2 energies were replaced by the corresponding κ-PT2 terms, evaluated for a fixed κ value, and then all four linear parameters were reoptimized to obtain the regularized double hybrids, κDSD-PBEP86-D3BJ and κxDSD_75_-PBEP86-D3BJ. Including κ = ∞ (i.e., unregularized GLPT2), we calculated WTMAD2 for 17 κ values ranging from 0.9 to 10. For both κDSD-PBEP86-D3BJ and κxDSD_75_-PBEP86-D3BJ, WTMAD2 decreases with an increase in κ, and beyond κ = 4.0, WTMAD2 approaches WTMAD2_κ=∞_ (see Figure S1). Interestingly, for κDSD-PBEP86-D3BJ, we observe a very shallow dip near κ = 4.0, but WTMAD2_κ=4.0_ is only 0.03 kcal/mol lower than WTMAD2_κ=∞_.

Having initially scanned κ-space for one gDH and one xDH, we then used full GMTKN55 for the final parametrization of the κ-regularized DSD functionals. For this purpose, we selected nine κ values (i.e., κ = 1.1, 1.45, 2.0, 2.2, 2.5, 3.0, 3.5, 4.0, and ∞) and three different exchange correlation (XC) combinations: PBE-P86, PBE-PBE, and B88-LYP. Among the three regularized DSD (i.e., κDSD-PBEP86-D3BJ, κDSD-PBEPBE-D3BJ, and κDSD-BLYP-D3BJ) and one regularized xDSD (i.e., κxDSD_75_-PBBEP86-D3BJ) functionals, only κDSD-BLYP-D3BJ marginally benefits from the PT2 regularization. We obtained the lowest WTMAD2 (2.34 kcal/mol) for κDSD-BLYP-D3BJ at κ = 3.0, which is just 0.09 kcal/mol lower than the WTMAD2 of the unregularized counterpart, revDSD-BLYP-D3BJ (see Figure 1 and Table S1). [By way of perspective, in the Supporting Information of ref (57), we applied the Bayesian information criterion^62,63^ to see what reduction in WTMAD2 could be considered “decisive” and found 3.5%, or (in this context) ∼0.09 kcal/mol.] Now, partitioning each WTMAD2 into five major subcategories, we found that small-molecule thermochemistry, barrier heights, and intramolecular interactions do not benefit at all from PT2 regularization and κ = ∞ always offers the best performance (see Table S1). However, for the large-molecule reactions, all four κDSD functionals benefit from regularization, most prominently for κDSD-BLYP-D3BJ. Finally, for the intermolecular interactions, κ-regularization improved performance for κxDSD_75_-PBEP86-D3BJ, κDSD-PBEP86-D3BJ, and κDSD-PBEPBE-D3BJ. We obtained the best results at κ = 2.5 while using κxDSD_75_-PBEP86-D3BJ and at κ = 1.45 for κDSD-PBEP86-D3BJ and κDSD-PBEPBE-D3BJ. However, the performance of those three functionals deteriorates with an increase in κ. The unregularized variant wins the race for this subset when κDSD-BLYP-D3BJ is considered (see Figure S2 and Table S1).

**Figure 1:**
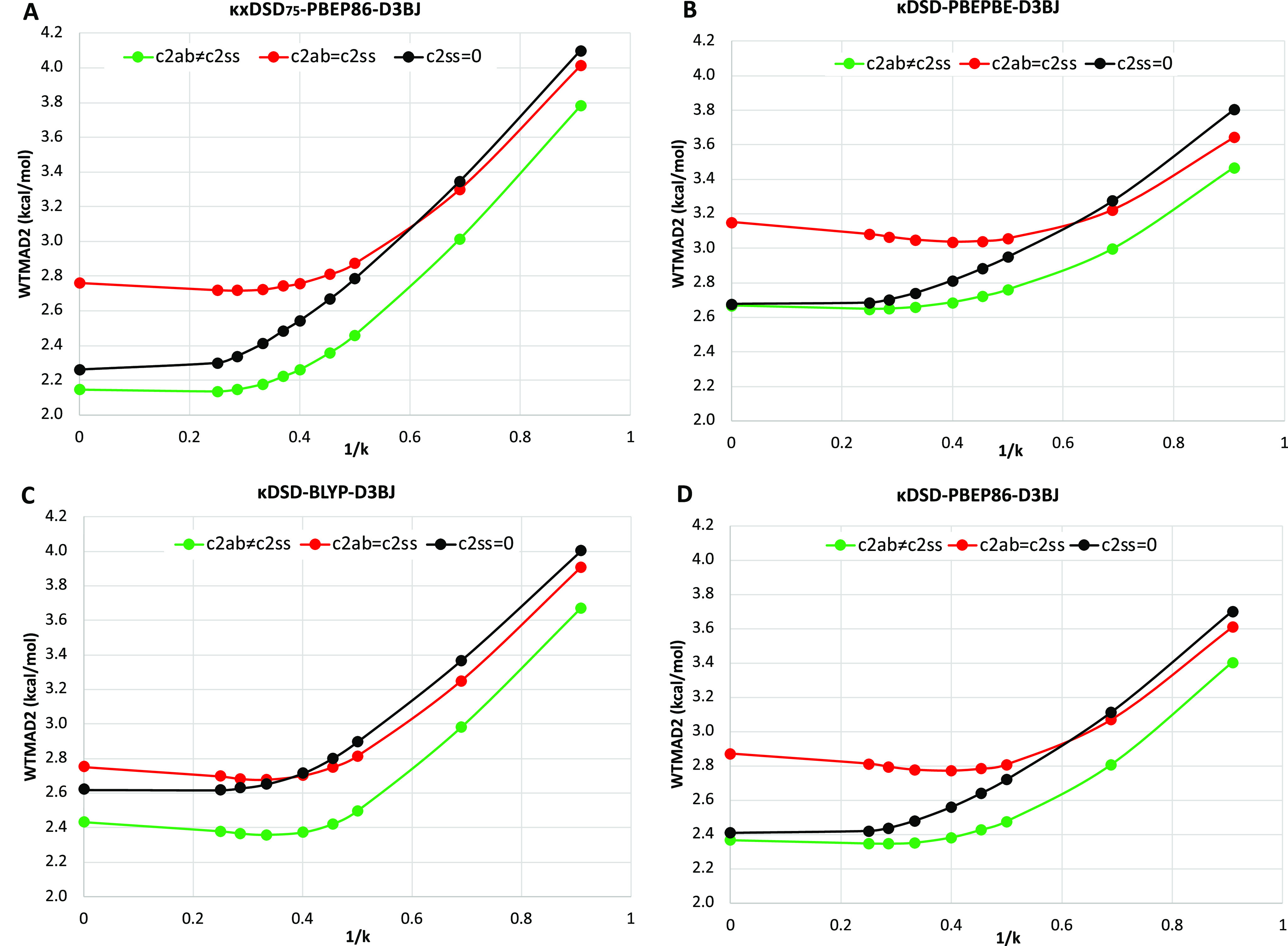
Dependence of total WTMAD2 (kcal/mol) on reciprocal κ for three variants of each of the regularized double-hybrid functionals (A) κxDSD_75_-PBBEP86-D3BJ, (B) κDSD-PBEPBE-D3BJ, (C) κDSD-BLYP-D3BJ, and (D) κDSD-PBEP86-D3BJ. Green solid lines represent the functionals where same- and opposite-spin coefficients are both optimized independently. The black lines are for the DOD variants, while the red lines represent the special case in which *c*_2ss_ = *c*_2ab_ (i.e., κDH-XC-D3BJ forms).

What if we allow only one degree of freedom between *c*_2ab_ and *c*_2ss_ but self-consistently reoptimize the remaining three parameters (i.e., *c*_C,DFT_, *c*_2ab_, and *s*_6_)? We considered two possibilities. The first is imposing the condition *c*_2ab_ = *c*_2ss_, i.e., simple double hybrids like B2PLYP,^64^ B2GPPLYP,^65^ PBE0-2,^66^ etc., as well as the combinatorially optimized ωB97M(2).^42^ The nomenclature, “DSD”, is no longer appropriate in this case and will be replaced by κDH-XC-D3BJ. The second option is to exclude same-spin correlation entirely (*c*_2ss_ = 0), also known as the DOD forms. These are of interest because they are amenable to reduced-scaling opposite-spin MP2 techniques like Laplace transform MP2 of Häser and Almlöf,^67^ which scales as Ο(N^4^) with system size, or the tensor hypercontraction approach of Song and Martinez,^68^ which scales as Ο(N^3^). As expected, constraining *c*_2ab_ = *c*_2ss_ always increases WTMAD2 over the κDSD forms. For both κDH-PBEPBE-D3BJ and κDH-PBEP86-D3BJ, we found the lowest WTMAD2 at κ_min_ = 2.5, corresponding to decreases of 0.11 and 0.10 kcal/mol, respectively, compared to their unregularized forms. For κDH-BLYP-D3BJ, the WTMAD2 gap between κ_min_ (κ value for which we obtain the minimum WTMAD2) and κ = ∞ is ∼0.08 kcal/mol. Interestingly, for the κDOD functionals, GLPT2 regularization does more harm than good, and the unregularized forms always offer the best performance (see Figure 1).

The use of the more modern D4^69,70^ dispersion correction instead of D3BJ for the regularized functionals does not affect any trends with respect to the regularizer (κ). For the PBE-PBE and PBE-P86 exchange-correlation combinations, the WTMAD2 gaps between the D3BJ and D4 corrected forms increase gradually with an increase in κ. However, switching from D3BJ to D4 has no significant effect on the performance of the κxDSD_75_-PBEP86-D4 and κDSD-BLYP-D4 functionals (see Table S2). The same story is repeated for the κDOD and κxDOD functionals with D4 dispersion correction (see Table S3).

Thus far, for the κDSD functionals, we have used the same parameter for exact exchange as for their unregularized forms.^40,48^ Our next objective is to check whether the Hartree–Fock exchange prefactors of the unregularized forms are still optimal for the new regularized functionals. To answer this question, we have considered seven *c*_X,HF_ values ranging from 0.5 to 1.0, and the κDSD*_X_*-PBEP86-D3BJ functional together with its κDH, κDOD, and dispersion uncorrected variants (*X* represents the percentage of HF exchange used; only one joint set of electronic structure calculations is required for all variants).

It turns out that regularization in κDSD-PBEP86-D3BJ becomes gradually more beneficial as *c*_X,HF_ is decreased, with κ_min_ decreasing concomitantly. For example, we obtain the lowest WTMAD2 near κ = 1.67 when *c*_X,HF_ = 0.55, but when *c*_X,HF_ = 0.5, the κ_min_ decreases to 1.45 (see Figure 2). Now, splitting each WTMAD2 into five major subcategories, we found that regularization does more harm than good across the board for small-molecule thermochemistry. However, the extent of performance deterioration with respect to the κ values becomes less prominent as *c*_X,HF_ is decreased. Using a small κ value can severely harm the performance for barrier heights at higher percentages of HF exchange, but much less so at lower percentages. When κ = 1.1, the κDSD_50_-PBEP86-D3BJ functional marginally outperforms the unregularized variant. For large-molecule reactions, the κDSD-PBEP86-D3BJ functional is a better choice across the board compared to the revDSD-PBEP86-DBJ functional, and κ_min_ decreases gradually with an increase in *c*_X,HF_. The regularized forms with 69% and 66% Hartree–Fock exchange offer the best performance near κ = 3.0 and 2.0, respectively. For intramolecular interactions, the trends are largely the same as what we obtained for the small-molecule thermochemistry subsets. Finally, for intermolecular interactions, regularized forms always outperform the unregularized alternatives, and κ_min_ decreases with an increase in *c*_X,HF_. For this subset, κDSD_69_-PBEP86-D3BJ and κDSD_63_-PBEP86-D3BJ are the two best picks at κ = 1.45 and 1.33, respectively (for optimized parameters, the total WTMAD2 for full GMTKN55, and its decomposition into five major subsets, see Table S4).

**Figure 2:**
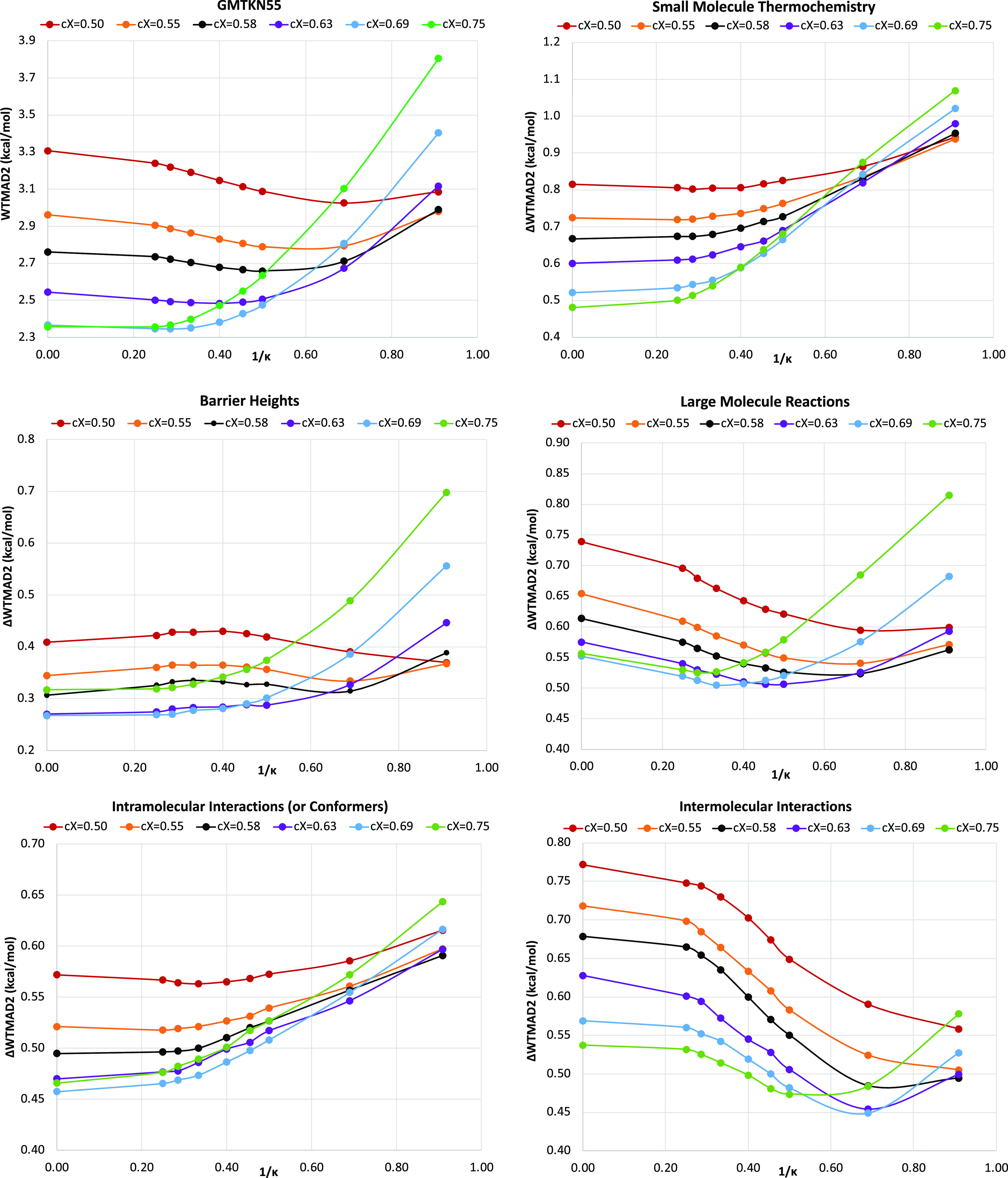
Dependence of total WTMAD2 (kcal/mol) and contribution (ΔWTMAD2 in kcal/mol) from five major subcategories on reciprocal κ for κDSD_*X*_-PBEP86-D3BJ. Six colors represent six different fractions of exact exchange (*c*_X_) ranging from 0.75 to 0.50.

The benefit in noncovalent interactions is not as prominent as that found by Shee et al.^39^ for pure MP2, which is expected because MP2 correlation in their case has a coefficient of unity while in a DHDF functional the MP2-like correlation (or PT2 correlation) is scaled down by a factor in the range of 0.3–0.5; hence, regularization will impact overall performance less. Additionally, HF exchange and PT2 correlation in a basis of KS orbitals are a different proposition from the same in a basis of HF orbitals.

As we found no material change from D3BJ to D4 in the previous section, we have decided not to explore that avenue for different *c*_X,HF_ values.

Now, what happens if we impose *c*_2ab_ = *c*_2ss_, i.e., a simple double hybrid rather than a DSD or DOD form? (As always, parameters are reoptimized self-consistently.) The results can be found in Figure S3 and Table S5. Even when *c*_X,HF_ = 0.75, we find a shallow WTMAD2 reduction (0.04 kcal/mol) at κ_min_; as *c*_X,HF_ is decreased, this “well” is deepened until it reaches 0.36 kcal/mol at *c*_X,HF_ = 0.50 (κ_min_ decreases in tandem with *c*_X,HF_). Among the five major subsets, at low *c*_X,HF_ values, the WTMAD2 component from noncovalent interactions (NCI) decreases as κ_min_ decreases, while this is detrimental to small-molecule thermochemistry and (at κ_min_ = 1.1–1.45) for barrier heights: the former tendency grows weaker, and the latter stronger, as *c*_X,HF_ is increased. We note that the ωB97M(2) combinatorially optimized range-separated double hybrid of Mardirossian and Head-Gordon^42^ has *c*_2ab_ = *c*_2ss_ and might hence benefit. (The way spin-component-scaled MP2 behaves differently from standard MP2 has been rationalized to some degree as approximate higher-order effects.^71,72^)

Interestingly, the behavior seen for the κDOD*_X_*-PBEP86-D3BJ functionals (i.e., when *c*_2ss_ = 0) is fairly similar, with lower percentages of HF exchange significantly benefiting from PT2 regularization. For example, the WTMAD2 “well” for κDOD_50_-PBEP86-D3BJ is 0.39 kcal/mol, but for κDOD_63_-PBEP86-D3BJ, this shrinks to just 0.04 kcal/mol. Except for small-molecule thermochemistry, we found the same trend as κDSD_*X*_-PBEP86-D3BJ for the remaining subsets. At small *c*_X,HF_ values, regularization seems to be slightly beneficial for the small-molecule thermochemistry subsets (see Figure S3 and Table S6).

For the sake of completeness, for dispersion-uncorrected κnoDispSD_*X*_-PBEP86 functionals, we likewise found that at higher fractions of HF exchange, κ_min_ approaches infinity (see Figure S3 and Table S7). In this case, obviously the balance is tipped more strongly toward high percentages of PT2 correlation (and of HF exchange, as the two are well-known^65^ to be linearly related) as long-range dispersion is not covered by anything else.

Thus far, we have used the same percentage of HF and semilocal exchange for orbital generation and final energy calculation. What happens when we use a different fraction of HF and DFT exchange for orbitals and final energies, e.g., PBE0P86 (i.e., 0.25HFx + 0.75PBEx + 1.0P86c) orbitals in κDSD_69_-PBEP86-D3BJ? These new DSD functionals are found not to benefit from PT2 regularization (see Table S8 and Figure S4). When WTMAD2 = 2.28 kcal/mol, the unregularized version, revDSD-PBEP86-D3BJ@PBE0P86, slightly outperforms the original revDSD-PBEP86-D3BJ (WTMAD2 = 2.38 kcal/mol): nearly all of that gain comes from RSE43 (radical-stabilization energies), due to substantially reduced spin contamination thanks to the smaller *c*_X,HF_ in the orbitals. If we constrain *c*_2ab_ = *c*_2ss_, the WTMAD2 gap between κ_min_ and κ = ∞ is 0.13 kcal/mol, marginally larger than what we obtained for κDH_69_-PBEP86-D3BJ (0.10 kcal/mol). κ_min_ also increases from 2.0 (for κDH_69_-PBEP86-D3BJ) to 3.0. For the κDOD variants of these new functionals, we saw the same trend that we did for the κDSD forms.

Next, we check the effect of PT2 regularization in three nonempirical double hybrids: SOS0-PBE0-2-D3BJ,^73,74^ SOS1-PBE-QIDH-D3BJ,^75,76^ and PBE0-DH-D3BJ.^1,77^ Benchmarking against GMTKN55, Mehta et al.^74^ reported that the first two among these three functionals are the best performers among the nonempirical DHDFs. Similar to what we found for κDOD_*X*_-PBEP86-D3BJ, employing regularization in the PT2 term does more harm than good for SOS0-PBE0-2-D3BJ and SOS1-PBE-QIDH-D3BJ (see Table S9). Unlike κDH_50_-PBEP86-D3BJ, regularized PT2 correlation offers no benefit for PBE0-DH-D3BJ. One reason the nonempirical double hybrid with 50% HF exchange sees no benefit may well be that the parameter for the PT2 correlation is 1/8 only; hence, it would not matter enough in the total WTMAD2.

Summing up an extensive survey of regularized DHDFs using the large and chemically diverse GMTKN55, we can conclude the following.

The benefits of PT2 regularization for intermolecular interactions and large-molecule reactions are negated by the losses for small-molecule thermochemistry, barrier heights, and conformer energies. Hence, κ-GLPT2 correlation causes no significant reduction in WTMAD2 compared to the unregularized revDSD-PBEP86-D3BJ, revDSD-PBE-D3BJ, and xDSD_75_-PBEP86-D3BJ functionals. However, the significantly better performance of κDSD-BLYP-D3BJ when κ = 2.2 for large-molecule reactions has enough impact on WTMAD2 that, overall, it marginally outperforms revDSD-BLYP-D3BJ. Replacing D3BJ with D4 dispersion does not affect those trends.

If we eliminate spin-component scaling (i.e., *c*_2ab_ = *c*_2ss_), the WTMAD2 gap between the κ_min_ and κ = ∞ (i.e., unregularized) forms of κ(*x*)DH-XC-D3BJ is more significant than that we obtained for the κ(*x*)DSD functionals. In contrast, for the κ(*x*)DOD forms, unregularized functionals always perform better.

Regularization of the GLPT2 terms in double hybrids is most helpful at lower percentages (e.g., 50%) of HF exchange. At higher percentages of HF exchange, the benefits for intermolecular interactions and large-molecule reactions are outweighed by the deterioration in the remaining three subsets. At lower percentages of HF exchange, the benefits are heightened and the deterioration is mitigated, hence, an overall beneficial effect. In special cases in which DHs with a small fraction of HF exchange might be more resilient (e.g., systems with strong static correlation or prone to severe spin contamination error), κ-regularized double hybrids will offer advantages over their unregularized counterparts.

## Supporting Information Available

Optimized parameters, total WTMAD2 and its division into major subsets for κDSD-PBEP86-D3BJ, κDSD-PBEPBE-D3BJ, κDSD-BLYP-D3BJ, κxDSD_75_-PBEP86-D3BJ, κDSD-PBEP86-D4, κDSD-PBEPBE-D4, κDSD-BLYP-D4, κxDSD_75_-PBEP86-D4, κDOD-PBEP86-D4, κDOD-PBEPBE-D4, κDOD-BLYP-D4, and κxDOD_75_-PBEP86-D4; optimized parameters, total WTMAD2, and its division into major subcategories for κDSD_*X*_-PBEP86-D3BJ, κDH_*X*_-PBEP86-D3BJ, κDOD_*X*_-PBEP86-D3BJ, and κnoDispSD_*X*_-PBEP86, where seven different fractions of exact exchange (*c*_X,HF_) ranging from 1.0 to 0.5 are considered; optimized parameters, total WTMAD2, and its decomposition into major subcategories for κDSD_69_-PBEP86-D3BJ and κDSD_69_-PBEPBE-D3BJ@PBE0P86; dependence of WTMAD2 (kcal/mol) on reciprocal κ for κDSD-PBEP86-D3BJ and κxDSD_75_-PBEP86-D3BJ; dependence of total WTMAD2 (kcal/mol) and contribution (ΔWTMAD2 in kcal/mol) from five major subcategories on reciprocal κ for κxDSD_75_-PBEP86-D3BJ, κDSD-PBEP86-D3BJ, κDSD-PBEPBE-D3BJ, κDSD-BLYP-D3BJ, and κxDSD_75_-PBEP86-D3BJ; dependence of WTMAD2 (kcal/mol) on reciprocal κ for κDSD-PBEP86-D3BJ, κDOD-PBEP86-D3BJ, κDH-PBEP86-D3BJ, and κnoDispSD-PBEP86 functionals [the six colors represent six different fractions of exact exchange (*c*_X_) ranging from 0.75 to 0.50]; dependence of total WTMAD2 (kcal/mol) on reciprocal κ for κDSD_69_-PBEP86-D3BJ@PBE0P86 and κDSD_69_-PBEP86-D3BJ (A), their κDH counterparts (B), κDOD variants (C), and dispersion-uncorrected forms (D); total WTMAD2 and its decomposition into major subcategories for selected nonempirical DHDFs; and QCHEM sample input for κDSD-BLYP-D3BJ with κ = 2.2 (PDF)
