# Supplementary material for: Do Double-Hybrid Functionals Benefit from Regularization in the PT2 Term? Observations from an Extensive Benchmark
Source: J Phys Chem Lett. 2022 Apr 13;13(15):3499–506. doi: 10.1021/acs.jpclett.2c00718 (PMC9036584; doi:10.1021/acs.jpclett.2c00718)
Supplement: Supplementary file 1 — jz2c00718_si_001.pdf [file jz2c00718_si_001.pdf]

Supporting Information for:

Do Double Hybrid Functionals Benefit from Regularization in the PT2 Term? Observations from an Extensive Benchmark

Golokesh Santra and Jan M.L. Martin*

Department of Molecular Chemistry and Materials Science, Weizmann Institute of Science, 7610001 Reḥovot, Israel.

Email: gershom@weizmann.ac.il

Table S1: Optimized parameters, total WTMAD2 and its decomposition into major subcategories for κDSD-PBEP86-D3BJ, κDSD-PBEPBE-D3BJ, κDSD-BLYP-D3BJ, and κxDSD$_{75}$-PBEP86-D3BJ. Heatmapping is from red (worst) via yellow to green (best). Thermo=Small Molecule Thermochemistry; Barrier=barrier heights; Large=reaction energies for large systems; Conf=conformer/intramolecular interactions; Intermol=intermolecular interactions; and NCI= Total Non-Covalent Interaction (Conformer/Intramolecular + Intermolecular).

	WTMAD2	Parameters[a]					ΔWTMAD2 (kcal/mol)					
**Functionals**	**(kcal/mol)**	$c_{C,DFT}$	c_{2ab}	c_{2ss}	s_6	κ	**Thermo**	**Barrier**	**Large**	**Conf**	**Intermol**	**NCI**
**κDSD-PBEP86-D3BJ ($c_{X,HF}$=0.69)**	3.404	0.1713	0.8220	0.3588	0.5073	1.1	1.021	0.557	0.683	0.617	0.527	1.144
	2.807	0.2339	0.7410	0.2935	0.4331	1.45	0.842	0.386	0.576	0.555	0.449	1.004
	2.477	0.3176	0.6534	0.2193	0.3953	2.0	0.665	0.301	0.520	0.508	0.482	0.990
	2.428	0.3397	0.6336	0.2003	0.3950	2.2	0.628	0.291	0.512	0.498	0.500	0.998
	2.383	0.3647	0.6148	0.1740	0.3960	2.5	0.589	0.281	0.507	0.486	0.519	1.006
	2.352	0.3906	0.5974	0.1474	0.3989	3.0	0.554	0.277	0.505	0.474	0.542	1.016
	2.346	0.4075	0.5864	0.1220	0.4111	3.5	0.543	0.270	0.513	0.469	0.552	1.021
	2.348	0.4164	0.5824	0.1085	0.4175	4.0	0.534	0.269	0.519	0.466	0.560	1.026
	2.367	0.4312	0.5761	0.0816	0.4339	∞	0.521	0.268	0.552	0.457	0.569	1.026
**κDSD-PBEPBE-D3BJ ($c_{X,HF}$=0.68)**	3.467	0.1708	0.8221	0.3865	0.5489	1.1	1.070	0.544	0.693	0.644	0.516	1.160
	2.996	0.2431	0.7297	0.3071	0.5075	1.45	0.906	0.409	0.599	0.619	0.463	1.082
	2.760	0.3305	0.6601	0.2026	0.5136	2.0	0.755	0.342	0.535	0.620	0.508	1.127
	2.722	0.3506	0.6391	0.1983	0.5037	2.2	0.726	0.337	0.522	0.612	0.526	1.137
	2.686	0.3760	0.6182	0.1790	0.5061	2.5	0.693	0.329	0.511	0.608	0.546	1.154
	2.659	0.4068	0.6076	0.1376	0.5206	3.0	0.658	0.320	0.507	0.606	0.568	1.174
	2.650	0.4239	0.5985	0.1161	0.5347	3.5	0.644	0.314	0.506	0.607	0.578	1.185
	2.648	0.4293	0.5933	0.1095	0.5394	4.0	0.636	0.313	0.507	0.608	0.583	1.191
	2.668	0.4518	0.5893	0.0661	0.5739	∞	0.640	0.299	0.532	0.610	0.586	1.197
**κDSD-BLYP-D3BJ ($c_{X,HF}$=0.71)**	3.670	0.2647	0.8008	0.3807	0.6462	1.1	0.977	0.607	0.697	0.642	0.746	1.389
	2.978	0.3479	0.7033	0.3226	0.5770	1.45	0.789	0.425	0.543	0.564	0.657	1.221
	2.494	0.4197	0.6303	0.2878	0.5245	2.0	0.640	0.309	0.434	0.513	0.598	1.111
	2.418	0.4387	0.6166	0.2724	0.5236	2.2	0.612	0.292	0.426	0.506	0.583	1.089
	2.370	0.4621	0.5905	0.2728	0.5094	2.5	0.584	0.279	0.434	0.493	0.579	1.072
	2.355	0.4900	0.5714	0.2478	0.5181	3.0	0.566	0.274	0.464	0.483	0.568	1.051
	2.364	0.5092	0.5585	0.2327	0.5219	3.5	0.556	0.275	0.493	0.474	0.566	1.040
	2.377	0.5168	0.5551	0.2196	0.5279	4.0	0.556	0.274	0.518	0.468	0.561	1.030
	2.430	0.5342	0.5467	0.1924	0.5489	∞	0.556	0.275	0.575	0.468	0.556	1.024
**κxDSD$_{75}$-PBEP86-D3BJ ($c_{X,HF}$=0.75)**	3.781	0.1118	0.9307	0.3960	0.4170	1.1	1.062	0.690	0.725	0.663	0.641	1.303
	3.011	0.1774	0.8383	0.3231	0.3412	1.45	0.864	0.467	0.593	0.570	0.518	1.088
	2.459	0.2553	0.7557	0.2471	0.3112	2.0	0.680	0.327	0.487	0.516	0.449	0.965
	2.358	0.2709	0.7405	0.2319	0.3067	2.2	0.642	0.303	0.465	0.506	0.442	0.948
	2.261	0.2878	0.7201	0.2253	0.2932	2.5	0.601	0.284	0.442	0.491	0.442	0.933
	2.178	0.3151	0.7066	0.1905	0.2922	3.0	0.552	0.265	0.438	0.470	0.453	0.923
	2.146	0.3328	0.6929	0.1738	0.2913	3.5	0.525	0.256	0.446	0.453	0.466	0.919
	2.135	0.3382	0.6850	0.1772	0.2829	4.0	0.510	0.257	0.450	0.446	0.472	0.918
	2.145	0.3590	0.6777	0.1389	0.3071	∞	0.494	0.250	0.487	0.432	0.481	0.914

[a]All results are with fixed a_1 = 0; s_8 =0; and a_2= 5.5 (for κDSD-PBEP86-D3BJ and κDSD-PBEPBE-D3BJ), 5.2 (for κDSD-BLYP-D3BJ), and 5.6 (for κxDSD$_{75}$-PBEP86-D3BJ).

Table S2: Optimized parameters, total WTMAD2 and its decomposition into major subcategories for κDSD-PBEP86-D4, κDSD-PBEPBE-D4, κDSD-BLYP-D4, and κxDSD$_{75}$-PBEP86-D4. Heatmapping is from red (worst) via yellow to green (best). Thermo=Small Molecule Thermochemistry; Barrier=barrier heights; Large=reaction energies for large systems; Conf=conformer/intramolecular interactions; Intermol=intermolecular interactions; and NCI= Total Non-Covalent Interaction (Conformer/Intramolecular + Intermolecular).

	WTMAD2	Parameters[a]							ΔWTMAD2 (kcal/mol)					
**Functionals**	**(kcal/mol)**	$c_{C,DFT}$	c_{2ab}	c_{2ss}	s_6	a_1	a_2	κ	Thermo	Barrier	Large	Conf	Intermol	NCI
	3.431	0.1687	0.8473	0.3131	0.6038	0.012	5.751	1.1	1.008	0.560	0.693	0.588	0.581	1.169
	2.796	0.2270	0.7527	0.2577	0.5507	0.158	5.000	1.45	0.847	0.387	0.581	0.494	0.487	0.980
	2.410	0.2919	0.6841	0.1822	0.5461	0.262	4.461	2	0.700	0.303	0.508	0.447	0.452	0.899
**κDSD-PBEP86-D4**	2.349	0.3153	0.6629	0.1645	0.5469	0.282	4.394	2.2	0.663	0.289	0.505	0.439	0.453	0.892
**($c_{X,HF}$=0.69)**	2.294	0.3440	0.6400	0.1444	0.5458	0.312	4.327	2.5	0.624	0.279	0.507	0.432	0.453	0.885
	2.252	0.3719	0.6229	0.1170	0.5483	0.360	4.096	3	0.588	0.275	0.512	0.418	0.459	0.877
	2.239	0.3882	0.6130	0.0995	0.5558	0.369	4.103	3.5	0.572	0.272	0.521	0.414	0.460	0.874
	2.233	0.4012	0.6069	0.0816	0.5657	0.375	4.099	4	0.564	0.267	0.532	0.410	0.462	0.871
	2.248	0.4224	0.5935	0.0566	0.5917	0.371	4.201	∞	0.545	0.260	0.573	0.406	0.463	0.869
	3.472	0.1540	0.8696	0.3069	0.6543	0.052	5.351	1.1	1.060	0.544	0.717	0.562	0.589	1.151
	2.924	0.2309	0.7709	0.2302	0.6350	0.180	4.752	1.45	0.904	0.407	0.620	0.492	0.502	0.994
	2.580	0.3246	0.6785	0.1603	0.6421	0.304	4.108	2	0.761	0.341	0.555	0.447	0.475	0.922
**κDSD-PBEPBE-D4**	2.521	0.3456	0.6604	0.1425	0.6501	0.311	4.089	2.2	0.732	0.331	0.540	0.444	0.474	0.918
**($c_{X,HF}$=0.68)**	2.462	0.3607	0.6437	0.1296	0.6568	0.350	3.849	2.5	0.708	0.327	0.519	0.432	0.476	0.908
	2.400	0.3955	0.6257	0.0953	0.6706	0.353	3.883	3	0.669	0.315	0.508	0.425	0.483	0.908
	2.369	0.4108	0.6181	0.0769	0.6852	0.359	3.796	3.5	0.651	0.312	0.499	0.423	0.485	0.908
	2.354	0.4184	0.6169	0.0608	0.6997	0.381	3.745	4	0.643	0.310	0.501	0.419	0.481	0.900
	2.363	0.4371	0.6111	0.0318	0.7226	0.384	3.756	∞	0.635	0.301	0.523	0.420	0.484	0.904
	3.745	0.2819	0.8015	0.3617	0.7394	-0.015	5.689	1.1	0.957	0.609	0.743	0.626	0.810	1.436
	3.027	0.3480	0.7151	0.3112	0.6836	0.020	5.577	1.45	0.776	0.425	0.574	0.549	0.702	1.251
	2.512	0.4207	0.6480	0.2549	0.6576	0.072	5.335	2	0.633	0.315	0.450	0.499	0.615	1.114
**κDSD-BLYP-D4**	2.430	0.4426	0.6250	0.2527	0.6486	0.095	5.227	2.2	0.601	0.300	0.437	0.490	0.603	1.092
**($c_{X,HF}$=0.71)**	2.370	0.4671	0.5978	0.2541	0.6379	0.112	5.163	2.5	0.573	0.286	0.435	0.483	0.592	1.075
	2.352	0.4823	0.5821	0.2372	0.6484	0.213	4.539	3	0.557	0.287	0.448	0.468	0.592	1.060
	2.351	0.5078	0.5654	0.2204	0.6528	0.157	4.897	3.5	0.544	0.282	0.475	0.466	0.584	1.050
	2.365	0.5135	0.5608	0.2145	0.6567	0.147	4.950	4	0.541	0.281	0.496	0.464	0.583	1.047
	2.428	0.5231	0.5578	0.1891	0.6808	0.156	4.878	∞	0.545	0.282	0.554	0.468	0.579	1.047
	3.768	0.1191	0.9210	0.4375	0.5258	-0.337	8.486	1.1	1.042	0.689	0.735	0.696	0.607	1.302
	2.997	0.1799	0.8374	0.3464	0.4116	-0.335	8.236	1.45	0.848	0.467	0.597	0.567	0.519	1.085
	2.456	0.2403	0.7745	0.2404	0.3934	0.072	5.702	2	0.698	0.330	0.484	0.479	0.465	0.944
**κxDSD$_{75}$-PBEP86-D4**	2.354	0.2600	0.7572	0.2241	0.3910	0.095	5.623	2.2	0.658	0.307	0.464	0.468	0.457	0.925
**($c_{X,HF}$=0.75)**	2.252	0.2864	0.7340	0.2013	0.3944	0.114	5.592	2.5	0.609	0.286	0.450	0.454	0.453	0.907
	2.177	0.3013	0.7203	0.1816	0.3985	0.331	4.212	3	0.573	0.273	0.434	0.429	0.468	0.897
	2.126	0.3220	0.7069	0.1637	0.4057	0.190	5.193	3.5	0.544	0.259	0.441	0.430	0.452	0.882
	2.112	0.3348	0.6972	0.1518	0.4114	0.191	5.203	4	0.529	0.254	0.449	0.423	0.457	0.880
	2.118	0.3517	0.6623	0.1168	0.4246	0.283	4.720	∞	0.508	0.252	0.493	0.409	0.457	0.866

[a]All results are with fixed c_{ATM} = 1 and s_8 =0.

Table S3: Optimized parameters, total WTMAD2 and its decomposition into major subcategories for κDOD-PBEP86-D4, κDOD-PBEPBE-D4, κDOD-BLYP-D4, and κxDOD$_{75}$-PBEP86-D4. Heatmapping is from red (worst) via yellow to green (best). Thermo=Small Molecule Thermochemistry; Barrier=barrier heights; Large=reaction energies for large systems; Conf=conformer/intramolecular interactions; Intermol=intermolecular interactions; and NCI= Total Non-Covalent Interaction (Conformer/Intramolecular + Intermolecular).

	WTMAD2	Parameters[a]							ΔWTMAD2 (kcal/mol)					
**Functionals**	**(kcal/mol)**	$c_{C,DFT}$	c_{2ab}	c_{2ss}	s_6	a_1	a_2	κ	Thermo	Barrier	Large	Conf	Intermol	NCI
	3.632	0.2174	0.9390	0.0	0.7101	0.001	5.570	1.1	1.002	0.655	0.777	0.572	0.627	1.199
	3.004	0.2653	0.8332	0.0	0.6707	0.124	4.927	1.45	0.856	0.442	0.676	0.495	0.536	1.031
	2.585	0.3200	0.7413	0.0	0.6408	0.247	4.403	2	0.738	0.318	0.602	0.455	0.473	0.927
**κDOD-PBEP86-D4**	2.501	0.3363	0.7187	0.0	0.6350	0.264	4.345	2.2	0.710	0.300	0.580	0.444	0.468	0.911
**($c_{X,HF}$=0.69)**	2.419	0.3604	0.6921	0.0	0.6251	0.293	4.225	2.5	0.677	0.285	0.560	0.428	0.470	0.898
	2.336	0.3892	0.6638	0.0	0.6043	0.320	4.194	3	0.639	0.271	0.550	0.411	0.465	0.876
	2.297	0.4013	0.6456	0.0	0.6076	0.352	4.033	3.5	0.618	0.267	0.544	0.402	0.467	0.869
	2.280	0.4093	0.6341	0.0	0.6139	0.357	4.024	4	0.604	0.264	0.543	0.400	0.469	0.869
	2.271	0.4301	0.6131	0.0	0.6158	0.344	4.243	∞	0.575	0.253	0.581	0.399	0.464	0.862
	3.664	0.2310	0.9378	0.0	0.7777	0.045	5.323	1.1	1.032	0.644	0.793	0.578	0.617	1.195
	3.084	0.2885	0.8294	0.0	0.7367	0.180	4.596	1.45	0.898	0.441	0.703	0.503	0.540	1.043
	2.697	0.3553	0.7302	0.0	0.7255	0.296	4.039	2	0.778	0.341	0.618	0.466	0.495	0.961
**κDOD-PBEPBE-D4**	2.616	0.3682	0.7134	0.0	0.7218	0.309	3.990	2.2	0.752	0.322	0.594	0.463	0.485	0.948
**($c_{X,HF}$=0.68)**	2.528	0.3868	0.6886	0.0	0.7231	0.344	3.822	2.5	0.722	0.306	0.569	0.450	0.481	0.931
	2.441	0.4059	0.6624	0.0	0.7263	0.347	3.921	3	0.690	0.294	0.539	0.436	0.482	0.918
	2.398	0.4179	0.6463	0.0	0.7358	0.381	3.745	3.5	0.672	0.289	0.519	0.437	0.481	0.918
	2.375	0.4240	0.6383	0.0	0.7380	0.397	3.592	4	0.662	0.291	0.517	0.425	0.480	0.905
	2.369	0.4465	0.6175	0.0	0.7410	0.377	3.770	∞	0.641	0.289	0.531	0.423	0.485	0.908
	3.996	0.3470	0.9431	0.0	0.8170	-0.013	5.545	1.1	1.058	0.732	0.779	0.609	0.819	1.428
	3.323	0.3884	0.8559	0.0	0.7746	0.004	5.498	1.45	0.916	0.521	0.631	0.549	0.707	1.255
	2.846	0.4519	0.7574	0.0	0.7557	0.067	5.144	2	0.794	0.385	0.523	0.508	0.635	1.143
**κDOD-BLYP-D4**	2.751	0.4643	0.7379	0.0	0.7556	0.085	5.029	2.2	0.768	0.360	0.501	0.500	0.623	1.123
**($c_{X,HF}$=0.71)**	2.667	0.4801	0.7203	0.0	0.7512	0.100	4.966	2.5	0.750	0.336	0.483	0.495	0.604	1.099
	2.615	0.5029	0.6866	0.0	0.7592	0.133	4.777	3	0.718	0.321	0.490	0.484	0.601	1.085
	2.606	0.5334	0.6657	0.0	0.7469	0.107	4.996	3.5	0.708	0.314	0.510	0.479	0.595	1.074
	2.600	0.5442	0.6501	0.0	0.7530	0.127	4.892	4	0.692	0.311	0.527	0.477	0.593	1.070
	2.622	0.5548	0.6297	0.0	0.7661	0.146	4.779	∞	0.667	0.301	0.576	0.480	0.598	1.078
	4.055	0.1649	1.0712	0.0	0.6211	-0.090	6.100	1.1	1.072	0.815	0.835	0.647	0.686	1.333
	3.295	0.2213	0.9558	0.0	0.5503	-0.015	5.776	1.45	0.900	0.575	0.702	0.543	0.576	1.119
	2.721	0.2732	0.8599	0.0	0.5136	0.010	5.625	2	0.753	0.389	0.572	0.482	0.525	1.007
**κxDOD$_{75}$-PBEP86-D4**	2.597	0.2776	0.8399	0.0	0.5261	0.131	4.899	2.2	0.726	0.345	0.544	0.470	0.512	0.982
**($c_{X,HF}$=0.75)**	2.476	0.2994	0.8129	0.0	0.5133	0.144	4.917	2.5	0.691	0.319	0.515	0.454	0.497	0.952
	2.361	0.3175	0.7862	0.0	0.5160	0.286	4.201	3	0.657	0.288	0.497	0.437	0.482	0.919
	2.284	0.3431	0.7654	0.0	0.4943	0.138	5.143	3.5	0.624	0.276	0.477	0.426	0.483	0.908
	2.248	0.3538	0.7555	0.0	0.4903	0.169	5.064	4	0.610	0.267	0.480	0.419	0.473	0.892
	2.215	0.3695	0.7092	0.0	0.4865	0.199	4.952	∞	0.582	0.251	0.507	0.402	0.473	0.875

[a]All results are with fixed c_{ATM} = 1 and s_8 =0.

Table S4: Optimized parameters, total WTMAD2 and its decomposition into major subcategories for κDSD$_X$-PBEP86-D3BJ. Heatmapping is from red (worst) via yellow to green (best). Thermo=Small Molecule Thermochemistry; Barrier=barrier heights; Large=reaction energies for large systems; Conf=conformer/intramolecular interactions; Intermol=intermolecular interactions; and NCI= Total Non-Covalent Interaction (Conformer/Intramolecular + Intermolecular).

WTMAD2 (kcal/mol)	Parameters[a]							ΔWTMAD2 (kcal/mol)					
	$c_{X,HF}$	$c_{X,DFT}$	$c_{C,DFT}$	c_{2ab}	c_{2ss}	s_6	κ	Thermo	Barrier	Large	Conf	Intermol	NCI
3.085	0.50	0.50	0.3329	0.6187	0.0368	0.7936	1.1	0.943	0.370	0.599	0.616	0.558	1.174
3.024	0.50	0.50	0.4029	0.5240	0.0100	0.7458	1.45	0.864	0.390	0.595	0.586	0.590	1.176
3.087	0.50	0.50	0.4816	0.4590	-0.0633	0.7483	2.0	0.825	0.419	0.621	0.573	0.649	1.222
3.113	0.50	0.50	0.5014	0.4402	-0.0734	0.7499	2.2	0.817	0.425	0.629	0.568	0.674	1.242
3.146	0.50	0.50	0.5262	0.4156	-0.0825	0.7485	2.5	0.806	0.430	0.642	0.565	0.703	1.268
3.189	0.50	0.50	0.5481	0.3941	-0.0964	0.7596	3.0	0.805	0.429	0.663	0.563	0.730	1.293
3.218	0.50	0.50	0.5604	0.3818	-0.1007	0.7608	3.5	0.802	0.428	0.679	0.564	0.744	1.309
3.239	0.50	0.50	0.5671	0.3753	-0.1079	0.7673	4.0	0.806	0.422	0.695	0.567	0.748	1.315
3.307	0.50	0.50	0.5887	0.3581	-0.1237	0.7774	∞	0.816	0.409	0.739	0.572	0.772	1.344
2.978	0.55	0.45	0.2843	0.6742	0.1262	0.7143	1.1	0.938	0.367	0.571	0.597	0.505	1.102
2.795	0.55	0.45	0.3536	0.5687	0.0998	0.6675	1.45	0.836	0.334	0.540	0.561	0.524	1.085
2.790	0.55	0.45	0.4322	0.5045	0.0169	0.6654	2.0	0.763	0.356	0.549	0.539	0.583	1.122
2.807	0.55	0.45	0.4554	0.4877	-0.0026	0.6660	2.2	0.749	0.361	0.557	0.532	0.608	1.139
2.831	0.55	0.45	0.4799	0.4696	-0.0234	0.6678	2.5	0.737	0.365	0.570	0.527	0.633	1.160
2.863	0.55	0.45	0.5063	0.4471	-0.0423	0.6764	3.0	0.728	0.365	0.585	0.521	0.664	1.185
2.888	0.55	0.45	0.5233	0.4324	-0.0505	0.6777	3.5	0.720	0.365	0.599	0.519	0.685	1.204
2.905	0.55	0.45	0.5340	0.4236	-0.0593	0.6854	4.0	0.719	0.361	0.609	0.518	0.699	1.216
2.962	0.55	0.45	0.5554	0.4077	-0.0799	0.6975	∞	0.724	0.345	0.654	0.521	0.718	1.239
2.989	0.58	0.42	0.2537	0.6938	0.2093	0.6625	1.1	0.953	0.388	0.562	0.591	0.495	1.085
2.711	0.58	0.42	0.3202	0.5965	0.1598	0.6102	1.45	0.831	0.315	0.523	0.557	0.485	1.041
2.658	0.58	0.42	0.4059	0.5330	0.0658	0.6041	2.0	0.727	0.328	0.526	0.527	0.550	1.077
2.665	0.58	0.42	0.4288	0.5163	0.0390	0.6123	2.2	0.714	0.327	0.533	0.520	0.570	1.090
2.678	0.58	0.42	0.4546	0.4969	0.0185	0.6149	2.5	0.696	0.333	0.540	0.510	0.600	1.110
2.703	0.58	0.42	0.4848	0.4744	-0.0035	0.6184	3.0	0.680	0.335	0.552	0.500	0.635	1.135
2.722	0.58	0.42	0.5024	0.4615	-0.0188	0.6251	3.5	0.674	0.332	0.565	0.497	0.654	1.151
2.736	0.58	0.42	0.5130	0.4524	-0.0296	0.6346	4.0	0.674	0.326	0.575	0.497	0.665	1.161
2.761	0.58	0.42	0.5277	0.4438	-0.0446	0.6411	∞	0.667	0.307	0.614	0.495	0.679	1.173
3.116	0.63	0.37	0.2117	0.7499	0.2922	0.5855	1.1	0.980	0.447	0.593	0.597	0.499	1.096
2.672	0.63	0.37	0.2820	0.6549	0.2282	0.5260	1.45	0.819	0.327	0.526	0.546	0.454	1.001
2.506	0.63	0.37	0.3638	0.5852	0.1344	0.5112	2.0	0.690	0.287	0.506	0.517	0.505	1.023
2.490	0.63	0.37	0.3878	0.5691	0.1113	0.5111	2.2	0.661	0.288	0.506	0.506	0.528	1.033
2.484	0.63	0.37	0.4092	0.5497	0.0877	0.5189	2.5	0.646	0.284	0.510	0.499	0.545	1.044
2.488	0.63	0.37	0.4395	0.5319	0.0543	0.5260	3.0	0.624	0.283	0.522	0.486	0.572	1.058
2.494	0.63	0.37	0.4593	0.5192	0.0335	0.5386	3.5	0.612	0.280	0.530	0.478	0.594	1.072
2.501	0.63	0.37	0.4678	0.5118	0.0226	0.5470	4.0	0.610	0.275	0.540	0.477	0.601	1.078
2.544	0.63	0.37	0.4892	0.5012	-0.0043	0.5670	∞	0.601	0.270	0.575	0.470	0.628	1.098
3.404	0.69	0.31	0.1713	0.8220	0.3588	0.5073	1.1	1.021	0.557	0.683	0.617	0.527	1.144
2.808	0.69	0.31	0.2339	0.7410	0.2935	0.4331	1.45	0.842	0.386	0.576	0.555	0.449	1.004
2.476	0.69	0.31	0.3176	0.6534	0.2193	0.3953	2.0	0.665	0.301	0.520	0.508	0.482	0.990
2.428	0.69	0.31	0.3397	0.6336	0.2003	0.3950	2.2	0.628	0.291	0.512	0.498	0.500	0.998
2.383	0.69	0.31	0.3647	0.6148	0.1740	0.3960	2.5	0.589	0.281	0.507	0.486	0.519	1.006
2.352	0.69	0.31	0.3906	0.5974	0.1474	0.3989	3.0	0.554	0.277	0.505	0.474	0.542	1.016
2.346	0.69	0.31	0.4075	0.5864	0.1220	0.4111	3.5	0.543	0.270	0.513	0.469	0.552	1.021
2.348	0.69	0.31	0.4164	0.5824	0.1085	0.4175	4.0	0.534	0.269	0.519	0.466	0.560	1.026
2.367	0.69	0.31	0.4312	0.5761	0.0816	0.4339	∞	0.521	0.268	0.552	0.457	0.569	1.026
3.804	0.75	0.25	0.1295	0.9038	0.4260	0.4209	1.1	1.069	0.698	0.815	0.644	0.578	1.221
3.104	0.75	0.25	0.1922	0.8159	0.3559	0.3507	1.45	0.875	0.489	0.685	0.572	0.484	1.056
2.634	0.75	0.25	0.2699	0.7330	0.2826	0.3121	2.0	0.681	0.374	0.579	0.527	0.474	1.000
2.551	0.75	0.25	0.2888	0.7143	0.2639	0.3091	2.2	0.638	0.357	0.558	0.517	0.481	0.998
2.472	0.75	0.25	0.3118	0.6921	0.2496	0.2955	2.5	0.590	0.342	0.541	0.501	0.498	0.999

2.398	0.75	0.25	0.3335	0.6729	0.2281	0.2943	3.0	0.540	0.328	0.526	0.489	0.515	1.004
2.368	0.75	0.25	0.3498	0.6647	0.2053	0.3045	3.5	0.514	0.321	0.525	0.482	0.525	1.008
2.357	0.75	0.25	0.3601	0.6582	0.1917	0.3086	4.0	0.500	0.319	0.529	0.476	0.532	1.008
2.358	0.75	0.25	0.3755	0.6514	0.1638	0.3267	∞	0.481	0.317	0.556	0.466	0.537	1.003
6.195	1.00	0.00	-0.0601	1.2348	0.7766	0.0267	1.1	1.437	1.381	1.526	0.915	0.936	1.851
5.257	1.00	0.00	-0.0136	1.1566	0.6961	-0.0592	1.45	1.175	1.109	1.355	0.796	0.822	1.619
4.568	1.00	0.00	0.0745	1.0584	0.5820	-0.0650	2.0	0.952	0.970	1.182	0.729	0.735	1.464
4.429	1.00	0.00	0.0977	1.0359	0.5582	-0.0651	2.2	0.906	0.945	1.144	0.713	0.720	1.434
4.282	1.00	0.00	0.1172	1.0262	0.5181	-0.0548	2.5	0.855	0.922	1.099	0.705	0.701	1.406
4.141	1.00	0.00	0.1405	1.0178	0.4743	-0.0443	3.0	0.805	0.901	1.058	0.693	0.684	1.377
4.068	1.00	0.00	0.1580	1.0165	0.4339	-0.0315	3.5	0.779	0.894	1.034	0.685	0.678	1.363
4.029	1.00	0.00	0.1643	1.0162	0.4240	-0.0325	4.0	0.765	0.887	1.025	0.678	0.675	1.353
3.968	1.00	0.00	0.1710	1.0175	0.4025	-0.0194	∞	0.736	0.880	1.015	0.675	0.661	1.337

[a]All results are with fixed $a_1 = 0$; $s_8 = 0$; and $a_2 = 5.5$

Table S5: Optimized parameters, total WTMAD2 and its decomposition into major subcategories for κDH$_X$-PBEP86-D3BJ. Heatmapping is from red (worst) via yellow to green (best). Thermo=Small Molecule Thermochemistry; Barrier=barrier heights; Large=reaction energies for large systems; Conf=conformer/intramolecular interactions; Intermol=intermolecular interactions; and NCI= Total Non-Covalent Interaction (Conformer/Intramolecular + Intermolecular).

WTMAD2 (kcal/mol)	Parameters[a]							ΔWTMAD2 (kcal/mol)					
	$c_{X,HF}$	$c_{X,DFT}$	$c_{C,DFT}$	c_{2ab}	c_{2ss}	s_6	κ	Thermo	Barrier	Large	Conf	Intermol	NCI
3.399	0.50	0.50	0.2913	0.4083	0.4083	0.7315	1.1	1.220	0.377	0.569	0.645	0.588	1.232
3.366	0.50	0.50	0.3603	0.3288	0.3288	0.7018	1.45	1.136	0.403	0.591	0.621	0.616	1.237
3.454	0.50	0.50	0.4261	0.2578	0.2578	0.7110	2.0	1.108	0.421	0.640	0.616	0.669	1.285
3.489	0.50	0.50	0.4392	0.2437	0.2437	0.7159	2.2	1.108	0.424	0.655	0.618	0.683	1.301
3.533	0.50	0.50	0.4583	0.2260	0.2260	0.7244	2.5	1.108	0.426	0.670	0.618	0.711	1.329
3.590	0.50	0.50	0.4708	0.2090	0.2090	0.7396	3.0	1.126	0.419	0.697	0.625	0.723	1.348
3.629	0.50	0.50	0.4896	0.1980	0.1980	0.7354	3.5	1.115	0.429	0.705	0.626	0.753	1.379
3.654	0.50	0.50	0.4966	0.1924	0.1924	0.7373	4.0	1.117	0.432	0.716	0.628	0.763	1.391
3.730	0.50	0.50	0.5113	0.1776	0.1776	0.7525	∞	1.141	0.422	0.751	0.632	0.785	1.416
3.231	0.55	0.45	0.2366	0.4767	0.4767	0.6656	1.1	1.214	0.366	0.511	0.625	0.515	1.140
3.080	0.55	0.45	0.3158	0.3862	0.3862	0.6319	1.45	1.096	0.344	0.521	0.588	0.531	1.119
3.129	0.55	0.45	0.3825	0.3194	0.3194	0.6195	2.0	1.027	0.371	0.556	0.579	0.596	1.176
3.162	0.55	0.45	0.4001	0.3036	0.3036	0.6184	2.2	1.015	0.380	0.568	0.579	0.620	1.198
3.206	0.55	0.45	0.4222	0.2839	0.2839	0.6219	2.5	1.008	0.386	0.585	0.579	0.648	1.227
3.264	0.55	0.45	0.4446	0.2654	0.2654	0.6272	3.0	1.001	0.397	0.602	0.580	0.685	1.265
3.304	0.55	0.45	0.4598	0.2505	0.2505	0.6356	3.5	1.011	0.389	0.619	0.585	0.700	1.285
3.331	0.55	0.45	0.4690	0.2423	0.2423	0.6389	4.0	1.015	0.387	0.630	0.588	0.710	1.298
3.405	0.55	0.45	0.4880	0.2236	0.2236	0.6574	∞	1.040	0.374	0.664	0.591	0.736	1.326
3.214	0.58	0.42	0.2161	0.5090	0.5090	0.6271	1.1	1.211	0.375	0.507	0.621	0.499	1.121
2.981	0.58	0.42	0.2782	0.4333	0.4333	0.5729	1.45	1.082	0.342	0.491	0.583	0.485	1.067
2.979	0.58	0.42	0.3625	0.3509	0.3509	0.5668	2.0	0.991	0.341	0.521	0.563	0.564	1.127
3.005	0.58	0.42	0.3814	0.3326	0.3326	0.5703	2.2	0.981	0.343	0.533	0.562	0.586	1.147
3.044	0.58	0.42	0.4041	0.3153	0.3153	0.5666	2.5	0.962	0.358	0.545	0.560	0.620	1.180
3.098	0.58	0.42	0.4237	0.2988	0.2988	0.5688	3.0	0.951	0.372	0.562	0.561	0.652	1.214
3.137	0.58	0.42	0.4344	0.2875	0.2875	0.5749	3.5	0.957	0.372	0.579	0.565	0.665	1.230
3.165	0.58	0.42	0.4468	0.2769	0.2769	0.5799	4.0	0.961	0.368	0.591	0.567	0.679	1.246
3.214	0.58	0.42	0.4597	0.2626	0.2626	0.5968	∞	0.984	0.347	0.623	0.565	0.695	1.260
3.323	0.63	0.37	0.1741	0.5767	0.5767	0.5487	1.1	1.212	0.433	0.543	0.626	0.510	1.135
2.931	0.63	0.37	0.2364	0.4966	0.4966	0.4911	1.45	1.064	0.350	0.486	0.578	0.453	1.032
2.820	0.63	0.37	0.3131	0.4153	0.4153	0.4772	2.0	0.963	0.323	0.482	0.551	0.501	1.052
2.826	0.63	0.37	0.3396	0.3931	0.3931	0.4752	2.2	0.938	0.320	0.493	0.545	0.530	1.075
2.844	0.63	0.37	0.3594	0.3767	0.3767	0.4687	2.5	0.915	0.328	0.504	0.543	0.555	1.098
2.886	0.63	0.37	0.3796	0.3596	0.3596	0.4698	3.0	0.898	0.338	0.521	0.543	0.586	1.129
2.919	0.63	0.37	0.3992	0.3430	0.3430	0.4749	3.5	0.895	0.334	0.537	0.544	0.610	1.154
2.945	0.63	0.37	0.4078	0.3346	0.3346	0.4833	4.0	0.897	0.333	0.547	0.543	0.625	1.168
3.015	0.63	0.37	0.4220	0.3220	0.3220	0.4906	∞	0.900	0.339	0.586	0.543	0.647	1.190
3.609	0.69	0.31	0.1296	0.6531	0.6531	0.4548	1.1	1.234	0.533	0.646	0.645	0.551	1.196
3.070	0.69	0.31	0.1875	0.5744	0.5744	0.3849	1.45	1.059	0.406	0.554	0.584	0.466	1.050
2.808	0.69	0.31	0.2703	0.4911	0.4911	0.3582	2.0	0.926	0.355	0.500	0.545	0.482	1.027
2.784	0.69	0.31	0.2881	0.4737	0.4737	0.3575	2.2	0.902	0.352	0.495	0.540	0.495	1.036
2.773	0.69	0.31	0.3064	0.4567	0.4567	0.3543	2.5	0.877	0.353	0.493	0.536	0.514	1.050
2.779	0.69	0.31	0.3290	0.4370	0.4370	0.3547	3.0	0.852	0.358	0.497	0.530	0.542	1.072
2.796	0.69	0.31	0.3418	0.4259	0.4259	0.3586	3.5	0.840	0.363	0.505	0.527	0.561	1.088
2.814	0.69	0.31	0.3493	0.4196	0.4196	0.3579	4.0	0.834	0.368	0.516	0.526	0.571	1.097
2.872	0.69	0.31	0.3661	0.4073	0.4073	0.3629	∞	0.824	0.378	0.550	0.522	0.600	1.121
4.004	0.75	0.25	0.0607	0.7559	0.7559	0.3614	1.1	1.267	0.655	0.789	0.689	0.603	1.292
3.363	0.75	0.25	0.1295	0.6625	0.6625	0.2861	1.45	1.075	0.499	0.675	0.614	0.501	1.115
2.989	0.75	0.25	0.2170	0.5721	0.5721	0.2505	2.0	0.920	0.431	0.583	0.566	0.489	1.055
2.933	0.75	0.25	0.2425	0.5476	0.5476	0.2516	2.2	0.894	0.422	0.565	0.554	0.497	1.052
2.888	0.75	0.25	0.2650	0.5300	0.5300	0.2371	2.5	0.860	0.419	0.552	0.545	0.512	1.057

2.863	0.75	0.25	0.2874	0.5096	0.5096	0.2404	3.0	0.834	0.420	0.543	0.539	0.527	1.066
2.864	0.75	0.25	0.3019	0.4990	0.4990	0.2329	3.5	0.816	0.425	0.546	0.535	0.542	1.077
2.870	0.75	0.25	0.3070	0.4934	0.4934	0.2359	4.0	0.810	0.429	0.550	0.533	0.548	1.081
2.900	0.75	0.25	0.3190	0.4846	0.4846	0.2384	∞	0.796	0.442	0.575	0.523	0.564	1.087
6.317	1.00	0.00	-0.1264	1.1149	1.1149	-0.0507	1.1	1.567	1.290	1.541	0.955	0.965	1.919
5.438	1.00	0.00	-0.0722	1.0278	1.0278	-0.1395	1.45	1.306	1.062	1.395	0.849	0.826	1.675
4.858	1.00	0.00	0.0222	0.9244	0.9244	-0.1831	2.0	1.119	0.967	1.259	0.769	0.745	1.513
4.758	1.00	0.00	0.0404	0.9010	0.9010	-0.1835	2.2	1.095	0.952	1.222	0.751	0.739	1.490
4.665	1.00	0.00	0.0617	0.8795	0.8795	-0.1840	2.5	1.063	0.942	1.196	0.741	0.723	1.464
4.585	1.00	0.00	0.0832	0.8599	0.8599	-0.1978	3.0	1.034	0.936	1.165	0.722	0.728	1.450
4.553	1.00	0.00	0.0957	0.8474	0.8474	-0.1952	3.5	1.027	0.935	1.152	0.716	0.724	1.439
4.540	1.00	0.00	0.1008	0.8398	0.8398	-0.1857	4.0	1.027	0.937	1.146	0.715	0.716	1.431
4.534	1.00	0.00	0.1075	0.8306	0.8306	-0.1746	∞	1.024	0.943	1.150	0.713	0.705	1.417

[a]All results are with fixed $a_1 = 0$; $s_8 = 0$; and $a_2 = 5.5$

Table S6: Optimized parameters, total WTMAD2 and its decomposition into major subcategories for κDOD$_X$-PBEP86-D3BJ. Heatmapping is from red (worst) via yellow to green (best). Thermo=Small Molecule Thermochemistry; Barrier=barrier heights; Large=reaction energies for large systems; Conf=conformer/intramolecular interactions; Intermol=intermolecular interactions; and NCI= Total Non-Covalent Interaction (Conformer/Intramolecular + Intermolecular).

WTMAD2 (kcal/mol)	Parameters[a]							ΔWTMAD2 (kcal/mol)					
	$c_{X,HF}$	$c_{X,DFT}$	$c_{C,DFT}$	c_{2ab}	c_{2ss}	s_6	κ	Thermo	Barrier	Large	Conf	Intermol	NCI
3.090	0.50	0.50	0.3449	0.6243	0.0	0.8050	1.1	0.932	0.371	0.610	0.616	0.562	1.178
3.024	0.50	0.50	0.4044	0.5270	0.0	0.7511	1.45	0.864	0.388	0.597	0.586	0.589	1.176
3.099	0.50	0.50	0.4697	0.4360	0.0	0.7209	2.0	0.819	0.438	0.613	0.571	0.658	1.230
3.135	0.50	0.50	0.4842	0.4161	0.0	0.7173	2.2	0.814	0.448	0.622	0.570	0.680	1.250
3.180	0.50	0.50	0.5059	0.3915	0.0	0.7094	2.5	0.803	0.463	0.633	0.567	0.714	1.281
3.240	0.50	0.50	0.5234	0.3599	0.0	0.7185	3.0	0.820	0.458	0.657	0.569	0.736	1.305
3.284	0.50	0.50	0.5332	0.3424	0.0	0.7239	3.5	0.832	0.456	0.676	0.573	0.748	1.321
3.316	0.50	0.50	0.5407	0.3323	0.0	0.7242	4.0	0.837	0.457	0.688	0.575	0.758	1.333
3.417	0.50	0.50	0.5581	0.3024	0.0	0.7367	∞	0.876	0.442	0.735	0.582	0.784	1.365
3.021	0.55	0.45	0.3164	0.7010	0.0	0.7613	1.1	0.915	0.378	0.617	0.606	0.506	1.112
2.817	0.55	0.45	0.3754	0.5964	0.0	0.7131	1.45	0.829	0.332	0.570	0.564	0.522	1.087
2.792	0.55	0.45	0.4340	0.5133	0.0	0.6711	2.0	0.765	0.355	0.552	0.540	0.580	1.120
2.807	0.55	0.45	0.4552	0.4862	0.0	0.6651	2.2	0.749	0.362	0.557	0.531	0.609	1.140
2.834	0.55	0.45	0.4759	0.4620	0.0	0.6547	2.5	0.731	0.373	0.566	0.526	0.638	1.164
2.873	0.55	0.45	0.4992	0.4327	0.0	0.6524	3.0	0.717	0.382	0.579	0.522	0.674	1.196
2.906	0.55	0.45	0.5123	0.4139	0.0	0.6527	3.5	0.717	0.382	0.594	0.523	0.691	1.213
2.931	0.55	0.45	0.5204	0.4020	0.0	0.6572	4.0	0.718	0.383	0.603	0.522	0.705	1.227
3.020	0.55	0.45	0.5348	0.3731	0.0	0.6747	∞	0.752	0.368	0.647	0.527	0.726	1.253
3.080	0.58	0.42	0.3083	0.7364	0.0	0.7310	1.1	0.916	0.413	0.650	0.608	0.493	1.101
2.778	0.58	0.42	0.3642	0.6358	0.0	0.6782	1.45	0.814	0.323	0.590	0.557	0.495	1.053
2.677	0.58	0.42	0.4188	0.5541	0.0	0.6368	2.0	0.740	0.318	0.545	0.528	0.547	1.075
2.673	0.58	0.42	0.4352	0.5316	0.0	0.6318	2.2	0.722	0.322	0.541	0.521	0.568	1.089
2.679	0.58	0.42	0.4569	0.5043	0.0	0.6252	2.5	0.702	0.328	0.543	0.511	0.597	1.107
2.703	0.58	0.42	0.4837	0.4729	0.0	0.6178	3.0	0.680	0.335	0.552	0.500	0.635	1.135
2.725	0.58	0.42	0.4977	0.4545	0.0	0.6166	3.5	0.671	0.337	0.563	0.499	0.654	1.153
2.742	0.58	0.42	0.5038	0.4458	0.0	0.6181	4.0	0.668	0.338	0.573	0.500	0.664	1.164
2.786	0.58	0.42	0.5151	0.4258	0.0	0.6281	∞	0.677	0.320	0.609	0.500	0.680	1.179
3.305	0.63	0.37	0.2856	0.8226	0.0	0.6731	1.1	0.962	0.508	0.725	0.613	0.499	1.111
2.844	0.63	0.37	0.3336	0.7191	0.0	0.6219	1.45	0.823	0.354	0.639	0.568	0.460	1.028
2.601	0.63	0.37	0.3953	0.6234	0.0	0.5773	2.0	0.717	0.283	0.571	0.523	0.508	1.030
2.563	0.63	0.37	0.4111	0.6020	0.0	0.5675	2.2	0.694	0.279	0.556	0.511	0.523	1.035
2.525	0.63	0.37	0.4280	0.5782	0.0	0.5634	2.5	0.668	0.275	0.538	0.502	0.543	1.044
2.506	0.63	0.37	0.4525	0.5491	0.0	0.5533	3.0	0.640	0.276	0.535	0.484	0.572	1.056
2.504	0.63	0.37	0.4641	0.5326	0.0	0.5537	3.5	0.626	0.273	0.539	0.480	0.587	1.066
2.507	0.63	0.37	0.4724	0.5237	0.0	0.5519	4.0	0.615	0.274	0.543	0.475	0.600	1.075
2.544	0.63	0.37	0.4878	0.4993	0.0	0.5660	∞	0.600	0.271	0.575	0.471	0.628	1.098
3.702	0.69	0.31	0.2639	0.9147	0.0	0.6046	1.1	1.034	0.661	0.861	0.626	0.521	1.146
3.113	0.69	0.31	0.3128	0.8131	0.0	0.5470	1.45	0.875	0.460	0.739	0.570	0.468	1.039
2.720	0.69	0.31	0.3598	0.7291	0.0	0.4979	2.0	0.742	0.326	0.635	0.536	0.481	1.017
2.642	0.69	0.31	0.3733	0.7080	0.0	0.4861	2.2	0.711	0.304	0.610	0.526	0.490	1.017
2.561	0.69	0.31	0.3941	0.6790	0.0	0.4764	2.5	0.672	0.287	0.584	0.510	0.509	1.018
2.481	0.69	0.31	0.4193	0.6480	0.0	0.4651	3.0	0.630	0.273	0.561	0.485	0.532	1.017
2.439	0.69	0.31	0.4308	0.6281	0.0	0.4694	3.5	0.605	0.264	0.548	0.479	0.542	1.021
2.421	0.69	0.31	0.4362	0.6176	0.0	0.4725	4.0	0.592	0.259	0.548	0.476	0.547	1.023
2.410	0.69	0.31	0.4443	0.6047	0.0	0.4790	∞	0.567	0.253	0.569	0.466	0.556	1.022
4.201	0.75	0.25	0.2344	1.0233	0.0	0.5365	1.1	1.137	0.832	1.011	0.663	0.558	1.221
3.514	0.75	0.25	0.2837	0.9252	0.0	0.4543	1.45	0.969	0.598	0.869	0.579	0.499	1.077
2.992	0.75	0.25	0.3419	0.8202	0.0	0.4143	2.0	0.791	0.431	0.746	0.534	0.491	1.025
2.887	0.75	0.25	0.3507	0.8018	0.0	0.4105	2.2	0.752	0.398	0.711	0.532	0.494	1.026
2.770	0.75	0.25	0.3610	0.7832	0.0	0.4035	2.5	0.710	0.364	0.669	0.528	0.500	1.027

2.653	0.75	0.25	0.3796	0.7560	0.0	0.3921	3.0	0.662	0.337	0.629	0.512	0.513	1.025
2.583	0.75	0.25	0.3897	0.7394	0.0	0.3919	3.5	0.633	0.322	0.603	0.504	0.521	1.025
2.541	0.75	0.25	0.3978	0.7279	0.0	0.3916	4.0	0.615	0.315	0.588	0.496	0.526	1.023
2.492	0.75	0.25	0.4068	0.7124	0.0	0.3975	∞	0.586	0.302	0.591	0.483	0.530	1.013
6.871	1.00	0.00	0.1163	1.4450	0.0	0.2693	1.1	1.654	1.651	1.737	0.958	0.871	1.829
5.964	1.00	0.00	0.1691	1.3564	0.0	0.1453	1.45	1.453	1.345	1.564	0.812	0.790	1.601
5.211	1.00	0.00	0.1992	1.2903	0.0	0.1137	2.0	1.254	1.108	1.372	0.790	0.686	1.477
5.043	1.00	0.00	0.2085	1.2770	0.0	0.0980	2.2	1.213	1.057	1.324	0.771	0.678	1.449
4.858	1.00	0.00	0.2246	1.2563	0.0	0.0814	2.5	1.171	1.004	1.269	0.741	0.673	1.414
4.664	1.00	0.00	0.2389	1.2288	0.0	0.0755	3.0	1.111	0.965	1.202	0.716	0.671	1.386
4.558	1.00	0.00	0.2478	1.2093	0.0	0.0733	3.5	1.070	0.950	1.165	0.697	0.676	1.372
4.496	1.00	0.00	0.2547	1.2013	0.0	0.0698	4.0	1.056	0.938	1.145	0.684	0.673	1.357
4.395	1.00	0.00	0.2593	1.1853	0.0	0.0800	∞	1.012	0.927	1.110	0.678	0.668	1.346

[a]All results are with fixed $a_1 = 0$; $s_8 = 0$; and $a_2 = 5.5$

Table S7: Optimized parameters, total WTMAD2 and its decomposition into major subcategories for κnoDispSD$_X$-PBEP86. Heatmapping is from red (worst) via yellow to green (best). Thermo=Small Molecule Thermochemistry; Barrier=barrier heights; Large=reaction energies for large systems; Conf=conformer/intramolecular interactions; Intermol=intermolecular interactions; and NCI= Total Non-Covalent Interaction (Conformer/Intramolecular + Intermolecular).

WTMAD2	Parameters[a]							ΔWTMAD2 (kcal/mol)					
(kcal/mol)	$c_{X,HF}$	$c_{X,DFT}$	$c_{C,DFT}$	c_{2ab}	c_{2ss}	s_6	κ	Thermo	Barrier	Large	Conf	Intermol	NCI
5.721	0.50	0.50	0.3345	0.5009	0.6659	0.0	1.1	1.308	0.734	0.880	1.475	1.325	2.800
5.410	0.50	0.50	0.3764	0.4575	0.5529	0.0	1.45	1.204	0.869	0.848	1.325	1.163	2.488
5.342	0.50	0.50	0.4651	0.3712	0.4429	0.0	2.0	1.041	0.932	0.891	1.314	1.164	2.478
5.355	0.50	0.50	0.4813	0.3566	0.4270	0.0	2.2	1.020	0.959	0.907	1.308	1.162	2.469
5.389	0.50	0.50	0.5129	0.3385	0.3854	0.0	2.5	0.976	0.961	0.932	1.329	1.191	2.519
5.448	0.50	0.50	0.5429	0.3141	0.3504	0.0	3.0	0.930	0.956	0.968	1.365	1.228	2.593
5.497	0.50	0.50	0.5623	0.3010	0.3294	0.0	3.5	0.909	0.956	0.993	1.388	1.251	2.639
5.536	0.50	0.50	0.5746	0.2893	0.3242	0.0	4.0	0.900	0.964	1.010	1.401	1.262	2.662
5.663	0.50	0.50	0.6049	0.2585	0.2977	0.0	∞	0.857	0.926	1.080	1.475	1.326	2.801
5.260	0.55	0.45	0.2682	0.5895	0.7012	0.0	1.1	1.299	0.637	0.787	1.295	1.242	2.537
4.860	0.55	0.45	0.3296	0.5234	0.5665	0.0	1.45	1.138	0.722	0.749	1.165	1.086	2.251
4.747	0.55	0.45	0.4166	0.4404	0.4628	0.0	2.0	0.985	0.807	0.776	1.128	1.052	2.180
4.751	0.55	0.45	0.4392	0.4218	0.4374	0.0	2.2	0.948	0.821	0.791	1.132	1.059	2.191
4.771	0.55	0.45	0.4680	0.3989	0.4091	0.0	2.5	0.905	0.835	0.813	1.144	1.074	2.218
4.819	0.55	0.45	0.4961	0.3722	0.3848	0.0	3.0	0.862	0.846	0.846	1.166	1.099	2.265
4.865	0.55	0.45	0.5143	0.3537	0.3737	0.0	3.5	0.839	0.855	0.870	1.184	1.117	2.301
4.903	0.55	0.45	0.5299	0.3406	0.3600	0.0	4.0	0.819	0.847	0.889	1.208	1.140	2.348
5.020	0.55	0.45	0.5636	0.3129	0.3272	0.0	∞	0.778	0.815	0.949	1.275	1.203	2.478
5.040	0.58	0.42	0.2460	0.6318	0.7052	0.0	1.1	1.279	0.589	0.761	1.210	1.202	2.411
4.568	0.58	0.42	0.3045	0.5583	0.5757	0.0	1.45	1.097	0.643	0.700	1.081	1.048	2.129
4.417	0.58	0.42	0.3854	0.4835	0.4680	0.0	2.0	0.944	0.723	0.716	1.032	1.003	2.035
4.413	0.58	0.42	0.4144	0.4630	0.4406	0.0	2.2	0.906	0.741	0.729	1.033	1.004	2.037
4.426	0.58	0.42	0.4378	0.4406	0.4188	0.0	2.5	0.868	0.759	0.750	1.039	1.011	2.050
4.465	0.58	0.42	0.4693	0.4115	0.3947	0.0	3.0	0.821	0.774	0.780	1.058	1.032	2.090
4.507	0.58	0.42	0.4870	0.3927	0.3838	0.0	3.5	0.796	0.781	0.804	1.076	1.049	2.126
4.542	0.58	0.42	0.4986	0.3849	0.3709	0.0	4.0	0.783	0.783	0.823	1.090	1.064	2.154
4.600	0.58	0.42	0.5262	0.3599	0.3501	0.0	∞	0.737	0.754	0.872	1.130	1.107	2.237
4.782	0.63	0.37	0.1936	0.7027	0.7474	0.0	1.1	1.277	0.567	0.741	1.071	1.127	2.198
4.174	0.63	0.37	0.2611	0.6231	0.5981	0.0	1.45	1.060	0.567	0.641	0.941	0.964	1.905
3.934	0.63	0.37	0.3448	0.5467	0.4738	0.0	2.0	0.872	0.597	0.641	0.890	0.933	1.823
3.917	0.63	0.37	0.3670	0.5230	0.4569	0.0	2.2	0.835	0.615	0.650	0.888	0.930	1.818
3.913	0.63	0.37	0.3939	0.5060	0.4264	0.0	2.5	0.796	0.634	0.662	0.888	0.932	1.821
3.933	0.63	0.37	0.4309	0.4774	0.3956	0.0	3.0	0.745	0.646	0.687	0.905	0.950	1.855
3.963	0.63	0.37	0.4503	0.4603	0.3805	0.0	3.5	0.718	0.651	0.707	0.920	0.965	1.886
3.992	0.63	0.37	0.4572	0.4489	0.3814	0.0	4.0	0.710	0.664	0.724	0.927	0.968	1.895
4.090	0.63	0.37	0.4764	0.4312	0.3695	0.0	∞	0.695	0.677	0.781	0.948	0.988	1.937
4.618	0.69	0.31	0.1444	0.8036	0.7424	0.0	1.1	1.263	0.568	0.783	0.924	1.078	2.003
3.864	0.69	0.31	0.2065	0.7231	0.5995	0.0	1.45	1.031	0.516	0.646	0.785	0.887	1.672
3.480	0.69	0.31	0.2959	0.6381	0.4827	0.0	2.0	0.829	0.516	0.585	0.731	0.821	1.551
3.439	0.69	0.31	0.3145	0.6186	0.4659	0.0	2.2	0.791	0.526	0.585	0.724	0.813	1.537
3.408	0.69	0.31	0.3383	0.5953	0.4451	0.0	2.5	0.746	0.536	0.592	0.722	0.813	1.535
3.403	0.69	0.31	0.3680	0.5649	0.4214	0.0	3.0	0.693	0.537	0.612	0.731	0.829	1.560
3.415	0.69	0.31	0.3872	0.5464	0.4082	0.0	3.5	0.665	0.542	0.628	0.740	0.841	1.580
3.433	0.69	0.31	0.4049	0.5331	0.3922	0.0	4.0	0.641	0.535	0.643	0.753	0.861	1.614
3.509	0.69	0.31	0.4265	0.5149	0.3757	0.0	∞	0.618	0.540	0.693	0.773	0.885	1.658
4.592	0.75	0.25	0.1094	0.8806	0.7575	0.0	1.1	1.256	0.625	0.877	0.823	1.011	1.835
3.742	0.75	0.25	0.1750	0.8003	0.5994	0.0	1.45	0.999	0.512	0.723	0.681	0.828	1.509
3.224	0.75	0.25	0.2502	0.7250	0.4890	0.0	2.0	0.793	0.474	0.603	0.613	0.740	1.353
3.144	0.75	0.25	0.2680	0.7094	0.4661	0.0	2.2	0.751	0.473	0.582	0.604	0.733	1.337
3.071	0.75	0.25	0.2979	0.6836	0.4393	0.0	2.5	0.700	0.469	0.570	0.600	0.733	1.333

3.026	0.75	0.25	0.3250	0.6611	0.4161	0.0	3.0	0.654	0.472	0.573	0.597	0.732	1.328
3.018	0.75	0.25	0.3397	0.6422	0.4107	0.0	3.5	0.626	0.476	0.582	0.600	0.736	1.335
3.023	0.75	0.25	0.3481	0.6305	0.4089	0.0	4.0	0.612	0.479	0.591	0.602	0.739	1.341
3.068	0.75	0.25	0.3598	0.6192	0.3993	0.0	∞	0.595	0.488	0.633	0.603	0.749	1.353
6.195	1.00	0.00	-0.0676	1.2333	0.8180	0.0	1.1	1.442	1.359	1.535	0.913	0.948	1.860
5.266	1.00	0.00	-0.0069	1.1538	0.6469	0.0	1.45	1.168	1.133	1.350	0.828	0.786	1.615
4.585	1.00	0.00	0.0743	1.0693	0.5295	0.0	2.0	0.946	0.988	1.181	0.776	0.693	1.469
4.445	1.00	0.00	0.0925	1.0532	0.5064	0.0	2.2	0.901	0.961	1.141	0.765	0.678	1.443
4.296	1.00	0.00	0.1153	1.0329	0.4797	0.0	2.5	0.851	0.937	1.095	0.747	0.667	1.414
4.150	1.00	0.00	0.1438	1.0272	0.4267	0.0	3.0	0.801	0.915	1.048	0.719	0.667	1.386
4.074	1.00	0.00	0.1529	1.0229	0.4133	0.0	3.5	0.774	0.902	1.027	0.710	0.661	1.371
4.033	1.00	0.00	0.1600	1.0173	0.4073	0.0	4.0	0.759	0.896	1.018	0.702	0.658	1.360
3.971	1.00	0.00	0.1662	1.0183	0.3938	0.0	∞	0.731	0.886	1.008	0.691	0.655	1.346

[a]All results are with fixed $a_1 = 0$; $s_8 = 0$; and $a_2 = 5.5$

Table S8: Optimized parameters, total WTMAD2 and its decomposition into major subcategories for κDSD$_{69}$-PBEP86-D3BJ and κDSD$_{69}$-PBEPBE-D3BJ@PBE0P86. Heatmapping is from red (worst) via yellow to green (best). Thermo=Small Molecule Thermochemistry; Barrier=barrier heights; Large=reaction energies for large systems; Conf=conformer/intramolecular interactions; Intermol=intermolecular interactions; and NCI= Total Non-Covalent Interaction (Conformer/Intramolecular + Intermolecular).

Functionals	WTMAD2 (kcal/mol)	Parameters[a]					ΔWTMAD2 (kcal/mol)					
		$c_{C,DFT}$	c_{2ab}	c_{2ss}	s_6	κ	Thermo	Barrier	Large	Conf	Intermol	NCI
κDSD$_{69}$-PBEP86-D3BJ@ PBE0P86	3.821	0.1812	0.7392	0.2924	0.6121	1.1	1.136	0.638	0.727	0.680	0.641	1.321
	3.081	0.2537	0.6253	0.2330	0.5253	1.45	0.960	0.424	0.577	0.600	0.519	1.120
	2.594	0.3415	0.5054	0.1953	0.4683	2.0	0.800	0.305	0.479	0.547	0.463	1.010
	2.515	0.3597	0.4833	0.1880	0.4509	2.2	0.767	0.298	0.455	0.533	0.462	0.995
	2.436	0.3845	0.4579	0.1780	0.4331	2.5	0.726	0.297	0.425	0.516	0.473	0.989
	2.366	0.4305	0.4330	0.1417	0.4255	3.0	0.670	0.296	0.409	0.484	0.507	0.991
	2.326	0.4587	0.4165	0.1165	0.4325	3.5	0.644	0.290	0.401	0.465	0.526	0.991
	2.299	0.4757	0.4088	0.0977	0.4409	4.0	0.627	0.287	0.393	0.456	0.537	0.993
	2.276	0.5368	0.3811	0.0381	0.4883	∞	0.590	0.242	0.434	0.419	0.591	1.010
κDSD$_{69}$-PBEP86-D3BJ	3.404	0.1713	0.8220	0.3588	0.5073	1.1	1.021	0.557	0.683	0.617	0.527	1.144
	2.807	0.2339	0.7410	0.2935	0.4331	1.45	0.842	0.386	0.576	0.555	0.449	1.004
	2.477	0.3176	0.6534	0.2193	0.3953	2.0	0.665	0.301	0.520	0.508	0.482	0.990
	2.428	0.3397	0.6336	0.2003	0.3950	2.2	0.628	0.291	0.512	0.498	0.500	0.998
	2.383	0.3647	0.6148	0.1740	0.3960	2.5	0.589	0.281	0.507	0.486	0.519	1.006
	2.352	0.3906	0.5974	0.1474	0.3989	3.0	0.554	0.277	0.505	0.474	0.542	1.016
	2.346	0.4075	0.5864	0.1220	0.4111	3.5	0.543	0.270	0.513	0.469	0.552	1.021
	2.348	0.4164	0.5824	0.1085	0.4175	4.0	0.534	0.269	0.519	0.466	0.560	1.026
	2.367	0.4312	0.5761	0.0816	0.4339	∞	0.521	0.268	0.552	0.457	0.569	1.026

[a]All results are with fixed $a_1 = 0$; $s_8 = 0$; and $a_2 = 5.5$.

Table S9: Total WTMAD2 and its decomposition into major subcategories for selected nonempirical DHDFs. Thermo=Small Molecule Thermochemistry; Barrier=barrier heights; Large=reaction energies for large systems; Conf=conformer/intramolecular interactions; Intermol=intermolecular interactions; and NCI= Total Non-Covalent Interaction (Conformer/Intramolecular + Intermolecular).

Functionals	*κ*	*WTMAD2 (kcal/mol)*	*ΔWTMAD2 (kcal/mol)*					
			Thermo	*Barrier*	*Large*	*Conf*	*Intermol*	*NCI*
**PBE0-DH-D3BJ[1,2]**	1.1	5.745	1.469	0.629	1.282	1.096	1.270	2.366
	1.45	5.637	1.415	0.641	1.237	1.052	1.291	2.344
	2.0	5.545	1.357	0.657	1.183	1.027	1.320	2.347
	∞	5.439	1.273	0.698	1.100	1.003	1.365	2.368
**SOS1-PBE-QIDH-D3BJ[3,4]**	1.1	5.984	1.389	0.912	1.354	1.233	1.096	2.329
	1.45	5.233	1.223	0.760	1.234	1.024	0.993	2.016
	∞	3.838	0.874	0.456	0.873	0.768	0.867	1.635
**SOS0-PBE0-2-D3BJ[5,6]**	1.1	6.401	1.429	1.217	1.452	1.016	1.287	2.303
	1.45	5.347	1.214	0.994	1.293	0.825	1.021	1.846
	∞	3.461	0.736	0.561	0.846	0.672	0.647	1.320
**PBE0-DH[1]**	1.1	9.225	1.396	0.591	1.545	2.814	2.879	5.693
	1.45	8.822	1.330	0.568	1.495	2.678	2.750	5.428
	2.0	8.466	1.261	0.563	1.440	2.565	2.638	5.203
	∞	8.080	1.163	0.568	1.361	2.465	2.524	4.989
**SOS1-PBE-QIDH[3]**	1.1	9.611	1.374	1.011	1.534	2.638	3.055	5.693
	1.45	8.716	1.193	0.852	1.411	2.416	2.843	5.259
	∞	7.057	0.820	0.515	1.074	2.122	2.525	4.648
**SOS0-PBE0-2[5]**	1.1	9.686	1.392	1.345	1.577	2.409	2.964	5.373
	1.45	8.521	1.176	1.122	1.419	2.121	2.683	4.805
	∞	6.460	0.700	0.664	0.998	1.774	2.325	4.099

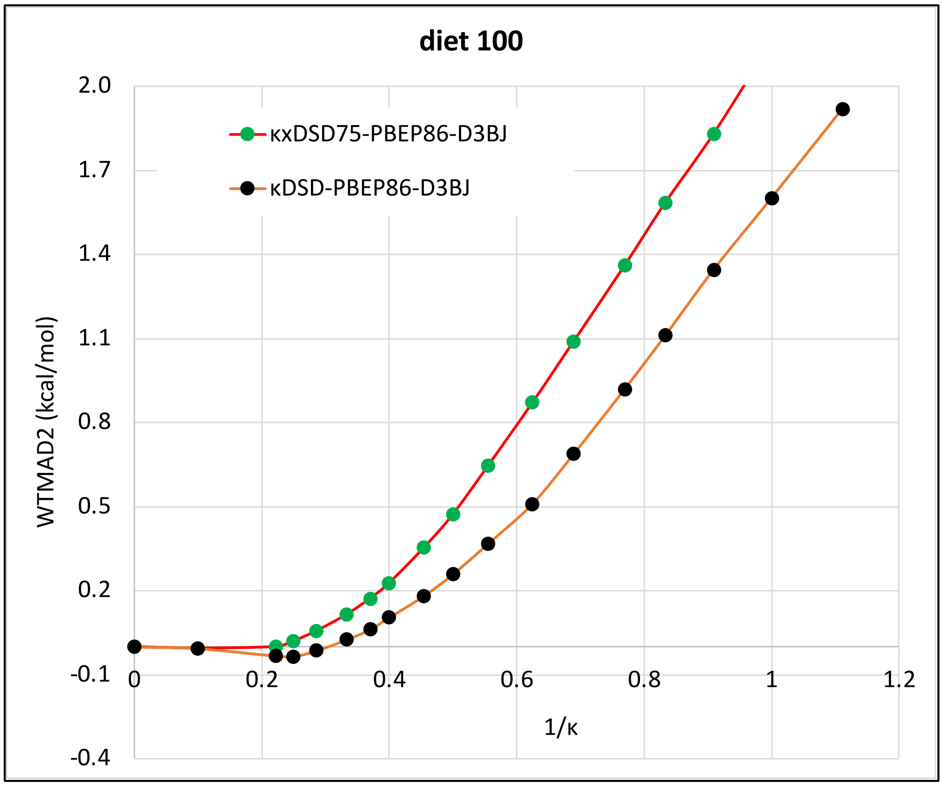

Figure S1: Dependence of WTMAD2 (kcal/mol) on reciprocal κ for κDSD-PBEP86-D3BJ and κxDSD$_{75}$-PBEP86-D3BJ.

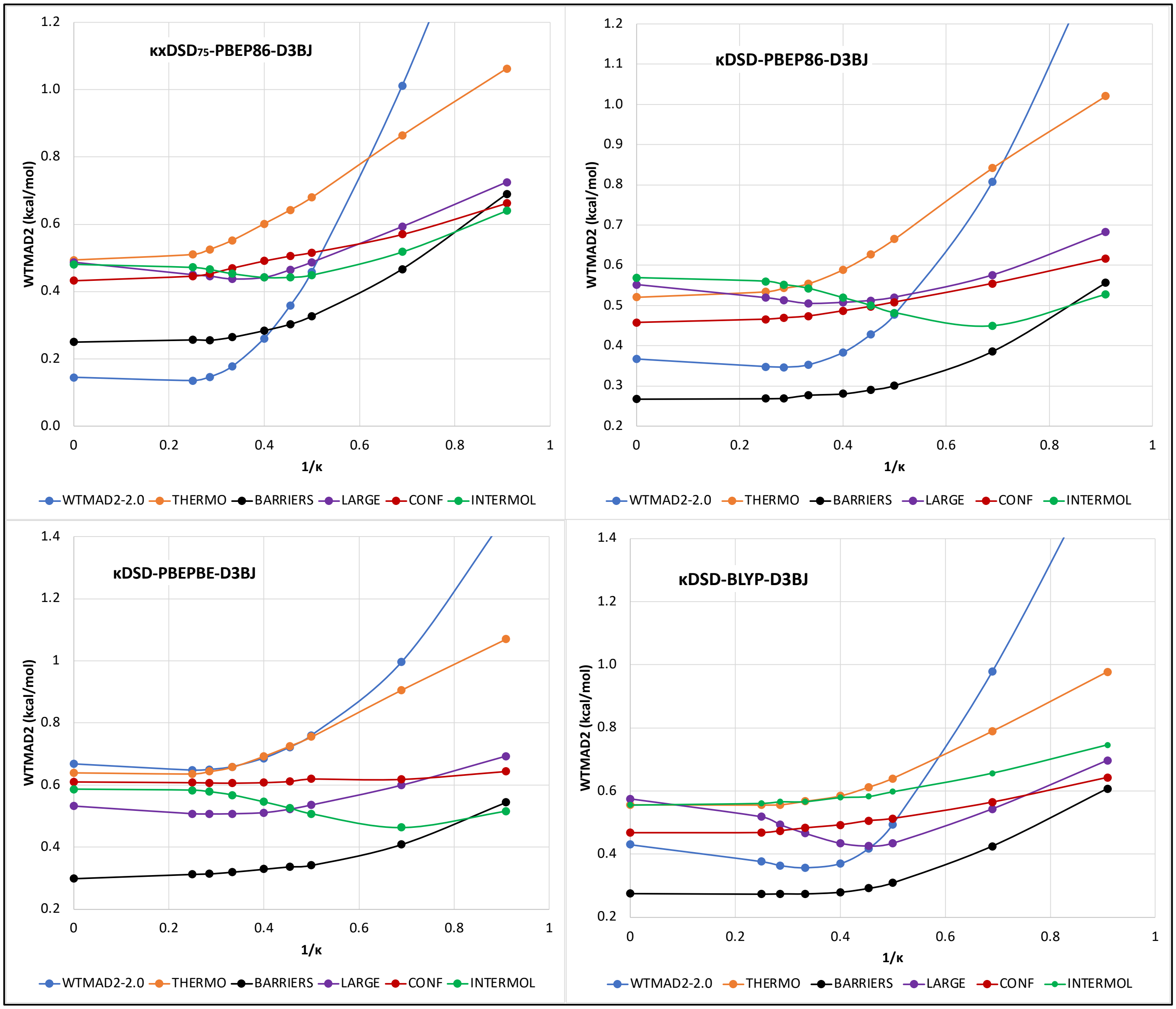

Figure S2: Dependence of total WTMAD2 (kcal/mol) and contribution (ΔWTMAD2 in kcal/mol) from five major subcategories on reciprocal κ for κxDSD$_{75}$-PBEP86-D3BJ, κDSD-PBEP86-D3BJ, κDSD-PBEPBE-D3BJ, κDSD-BLYP-D3BJ, and κxDSD$_{75}$-PBEP86-D3BJ. THERMO= Small Molecule Thermochemistry; BARRIER= barrier heights; LARGE= reaction energies for large systems; CONF= conformer/intramolecular interactions; and INTERMOL= intermolecular interactions.

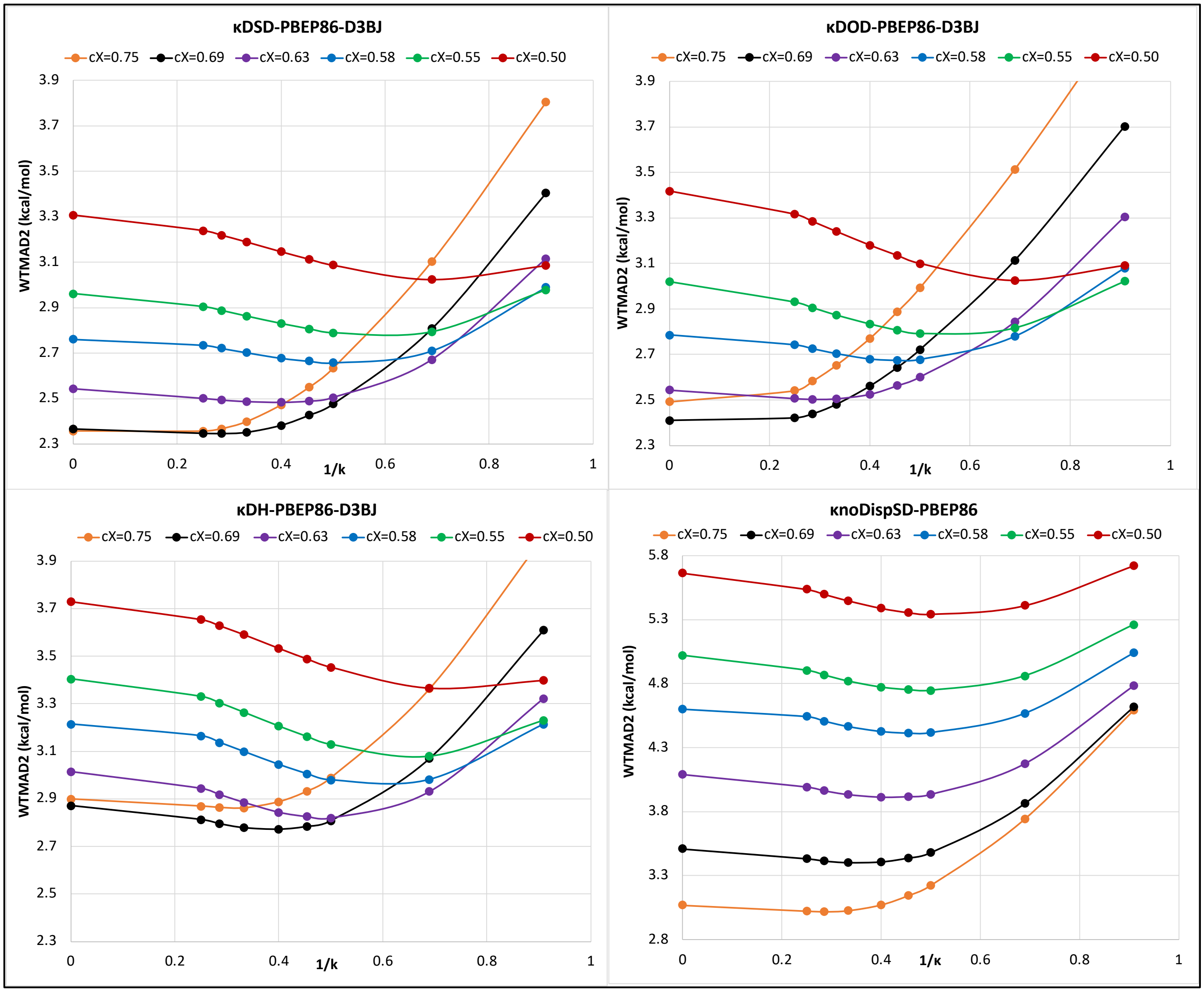

Figure S3: Dependence of WTMAD2 (kcal/mol) on reciprocal κ for κDSD-PBEP86-D3BJ, κDOD-PBEP86-D3BJ, κDH-PBEP86-D3BJ, and κnoDispSD-PBEP86 functionals. Six colors represent six different fractions of exact exchange (c_X) ranging from 0.75 to 0.50

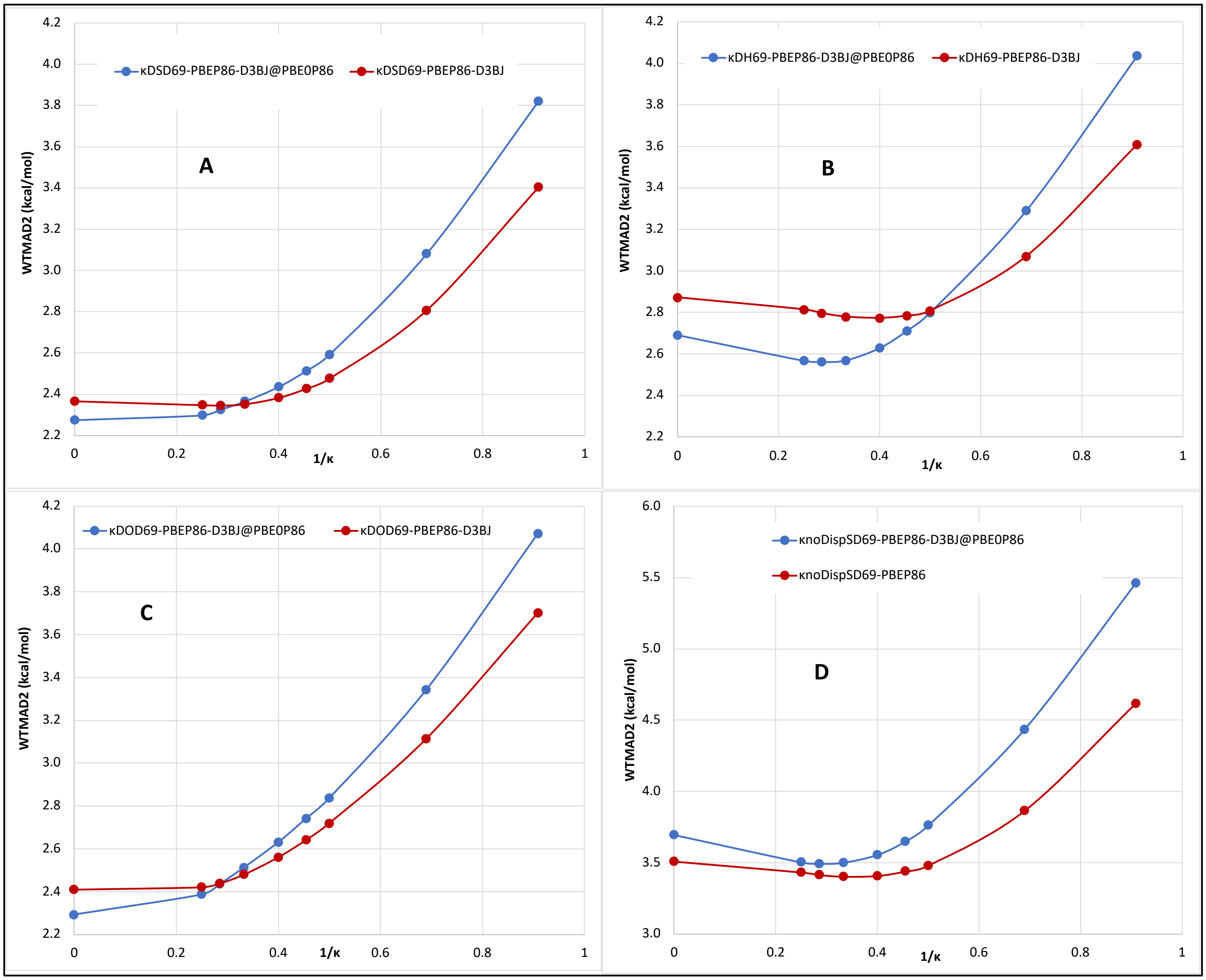

Figure S4: Dependence of total WTMAD2 (kcal/mol) on reciprocal κ for κDSD$_{69}$-PBEP86-D3BJ@PBE0P86 and κDSD$_{69}$-PBEP86-D3BJ (A); their κDH counterparts (B); κDOD variants (C); and the dispersion-*un*corrected forms (D).

Sample Input:

κDSD-BLYP-D3BJ (κ=2.2)

```
$rem
SYM_IGNORE            TRUE
SYMMETRY              false
BASIS                 def2-QZVPP
exchange              GEN
XC_GRID               3
ECP                   def2-ECP
MAX_SCF_CYCLES        1000
SCF_CONVERGENCE       7
THRESH                12
MEM_STATIC            2000
SET_ITER              100
MOLDEN_FORMAT         false
PRINT_ORBITALS        10
SCF_FINAL_PRINT       1
DFT_D                 D3_BJ
DFT_D3_S6             52360
DFT_D3_S8             0
DFT_D3_A2             520000
DFT_D3_A1             0
$end

$xc_functional
C LYP 0.4387
X HF  0.7100
X B88 0.2900
$end

$molecule
0 1
 C         0.000000000000      0.000000000000      0.000000000000
 O         0.000000000000      0.000000000000      1.131400000000
$end

@@@

$molecule
read
$end

$rem
   SCF_GUESS               read
   basis                   def2-QZVPP
   aux_basis               rimp2-def2-QZVPPD
   correlation             rimp2
   SCS                     3
   SSS_FACTOR              272400
   SOS_FACTOR              616600
   SYMMETRY                FALSE
   SYM_IGNORE              TRUE
   gen_scfman              true
   REGULARIZED_O2          2
   REG_VARIABLE            2200
```

```
   MEM_TOTAL               64000
   MEM_STATIC              2000
   n_frozen_core           FC
   SCF_CONVERGENCE         7
   THRESH                  12
   MAXSCF                  0
   ECP                     def2-ECP
$end
```

References:

(1) Brémond, E.; Adamo, C. Seeking for Parameter-Free Double-Hybrid Functionals: The PBE0-DH Model. *J. Chem. Phys.* **2011**, *135* (2), 024106. https://doi.org/10.1063/1.3604569.

(2) Goerigk, L.; Grimme, S. Double-Hybrid Density Functionals. *Wiley Interdiscip. Rev. Comput. Mol. Sci.* **2014**, *4* (6), 576–600. https://doi.org/10.1002/wcms.1193.

(3) Brémond, É.; Savarese, M.; Sancho-García, J. C.; Pérez-Jiménez, Á. J.; Adamo, C. Quadratic Integrand Double-Hybrid Made Spin-Component-Scaled. *J. Chem. Phys.* **2016**, *144* (12), 124104. https://doi.org/10.1063/1.4944465.

(4) Sancho-García, J. C.; Brémond; Savarese, M.; Pérez-Jiménez, A. J.; Adamo, C. Partnering Dispersion Corrections with Modern Parameter-Free Double-Hybrid Density Functionals. *Phys. Chem. Chem. Phys.* **2017**, *19* (21), 13481–13487. https://doi.org/10.1039/c7cp00709d.

(5) Alipour, M. Seeking for Spin-Opposite-Scaled Double-Hybrid Models Free of Fitted Parameters. *J. Phys. Chem. A* **2016**, *120* (20), 3726–3730. https://doi.org/10.1021/acs.jpca.6b03406.

(6) Mehta, N.; Casanova-Páez, M.; Goerigk, L. Semi-Empirical or Non-Empirical Double-Hybrid Density Functionals: Which Are More Robust? *Phys. Chem. Chem. Phys.* **2018**, *20* (36), 23175–23194. https://doi.org/10.1039/C8CP03852J.
